# Pre-activation of T cell immunity potentiates ferroptotic cell death through arachidonic acid hybridized nanovesicles

**DOI:** 10.1186/s12951-025-03797-x

**Published:** 2025-11-18

**Authors:** Qi Lyu, Chang Liu, Shaoyue Li, Dandan Shan, Hong Han, Liying Wang, Huixiong Xu

**Affiliations:** 1 Department of Ultrasound, Zhongshan Hospital, Institute of Ultrasound in Medicine and Engineering, Fudan University, Shanghai, 200032 P. R. China; 2 Department of Medical Ultrasound, Shanghai Frontiers Science Center of Nanocatalytic Medicine, School of Medicine, Shanghai Tenth People’s Hospital, Tongji University, Shanghai, 200072 P. R. China

**Keywords:** Ferroptosis, Outer membrane vesicles, T cell immunity, Arachidonic acid, Immune microenvironment

## Abstract

Ferroptotic tumor therapy is of highly significance to treat malignancies. Nevertheless, immune cells within the tumor immune microenvironment (TIME) are particularly susceptible to ferroptosis upon exposure to ferroptotic inducers, leading to the immunosuppression of TIME. In the present work, we report a dual activation strategy to initially pre-activate the T cells within TIME using genetically engineered cytolysin A-expressing outer membrane vesicles (OMVs) to enhance the interferon-γ (IFN-γ) production. We then deliver the homologous tumor cell membrane-hybridized liposomal nanovesicles containing arachidonic acid (MLipoAA) to the tumor cells for IFN-γ-potentiated ferroptosis. The combination treatment of OMVs and MLipoAA results in significant tumor destruction in colon CT26 tumor xenografts. This approach is also effective in combating tumor recurrence and metastasis. Mechanistically, the produced IFN-γ synergizes with arachidonic acid to trigger Acsl4-mediated tumor ferroptosis. The present work provides prominent immunopotentiations followed by selective tumor ferroptotic strategy with high effectiveness and biocompatibility, presenting a robust therapeutic strategy to conquer the immunosuppression drawback during in vivo ferroptotic therapy.

**Graphical Abstract:**

**Figure:**
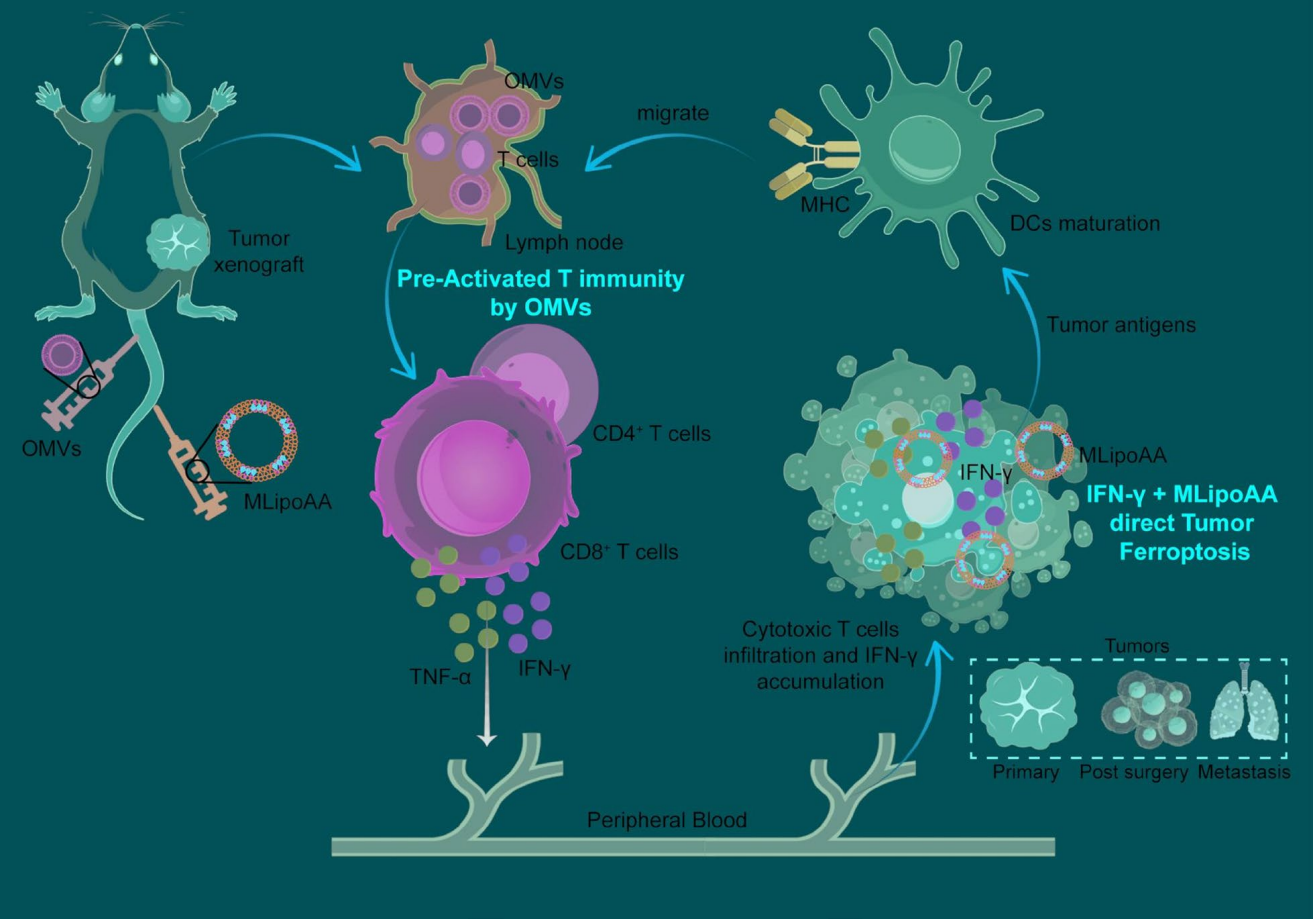
Pre-Activation of T cell Immunity Potentiates Tumor Ferroptotic Cell Death

**Supplementary Information:**

The online version contains supplementary material available at 10.1186/s12951-025-03797-x.

## Introduction

Ferroptosis has emerged as a cell death pathway, typically characterized by the iron-dependent regulated cell death originated from the accumulation of lipid peroxidation [1]. It has been widely implicated in various diseases such as cardiomyopathy [2], Alzheimer’s disease [3] and malignant tumor [4, 5]. Ferroptotic tumor therapy is commonly driven by the intratumoral accumulations of lipid peroxides, disrupting the cellular anti-oxidative system [6]. Upon ferroptosis, the compromised tumor cells could release several immunogenic patterns such as high-mobility group box 1, further stimulating the maturation of the dendritic cells for T cells priming [7, 8]. Such immunogenic ferroptotic therapy is therefore regarded as the most promising tumor therapeutic strategy among biomedical community. Medications of ferroptosis inducers such as imidazole ketone erastin (IKE), RSL3 have been developed and investigated extensively [9, 10]. Despite robust in vitro ferroptosis induction performance, the underlying negative effects during their in vivo applications are not fully discovered [11].

Tumor immune microenvironment (TIME) contains a variety of immune cells such as tumor associated macrophages, nature killer cells and T cells, etc [12, 13]. Systemic influences of ferroptosis on these immune cells within TIME are highly appealing to unveil the potentials for immunotherapy under practical conditions [14]. Recently, Kim and co-workers found that polymorphonuclear myeloid-derived suppressor cells (PMN-MDSCs), a major negative regulator of anti-tumor immunity, could spontaneously die by ferroptosis and release oxygenated lipids to limit the activity of T cells [15]. In addition, despite early ferroptotic tumor cells were found to release DAMPs, these antigens failed to promote dendritic cells (DCs) maturation due to the elevated gene expression of cyclooxygenase 2 (COX-2) [16] and prostaglandin E2 (PGE2) [17], which suppress the activity of anti-tumor T-helper cells and enhance immunosuppressive Tregs and MDSCs [18, 19]. In another research, pharmacologic screening reveals cytotoxic T lymphocytes (CTLs) are more sensitive to ferroptosis inducers than cancer cells [20]. These evidences suggest that during in vivo application of ferroptosis inducers, immune cells from TIME are much more vulnerable to ferroptosis induction, ultimately affecting both innate and adaptive immune responses [14, 21]. To induce immune activation during in vivo ferroptotic therapy, diverse immunopotentiation strategies have been developed. Intermittent dietary methionine intervention augments tumoral ferroptosis, sensitizes tumor cells against CD8 + T cell-mediated cytotoxicity, and synergizes with checkpoint blockade therapy [22]. Liu et al. synthesized ultrathin manganese-based layered double hydride nanosheets for GSH depletion and hydroxyl radical generation for tumor ferroptosis, which further cooperates with immunomodulatory property of encapsulated IFN-γ for tumor therapy [23]. Nevertheless, these immunopotentiation strategies still rely on the compromised immune cells upon ferroptosis challenge, constraining the overall therapeutic consequences. Collectively, conventional ferroptosis-inducing strategies inevitably trigger ferroptotic death in immune cells, thereby attenuating antitumor immune activation. Precise orchestration of the spatiotemporal dynamics of immune activation and ferroptosis induction could enable a more efficient and safer modality for tumor immunoactivation therapy.

In the present work, we propose a dual activation strategy to initially pre-activate the T cells within TIME using genetically cytolysin A engineered outer membrane vesicles (OMVs) to potentiate the interferon-γ (IFN-γ) production. We then deliver the homologue tumor cell membrane hybridized liposomal nanovesicles containing arachidonic acid (MLipoAA) to the tumor cells for IFN-γ-potentiated ferroptosis induction. The strategy not only maximally potentiates the anti-tumor immunities for tumor destruction, but also avoids the ferroptosis induction against the immune cells. By temporally adjusted combinational administration of OMVs and MLipoAA, effective in vivo ferroptotic therapy against subcutaneous CT26 colon xenografts could be achieved with high biocompatibility (Scheme 1). The promises of such therapeutics in treatments against tumor recurrence and metastasis have also been evaluated, illuminating the significances of pre-activation of T cell immunity IFN-γ-potentiated in vivo ferroptotic therapy.

**Fig. 1:**
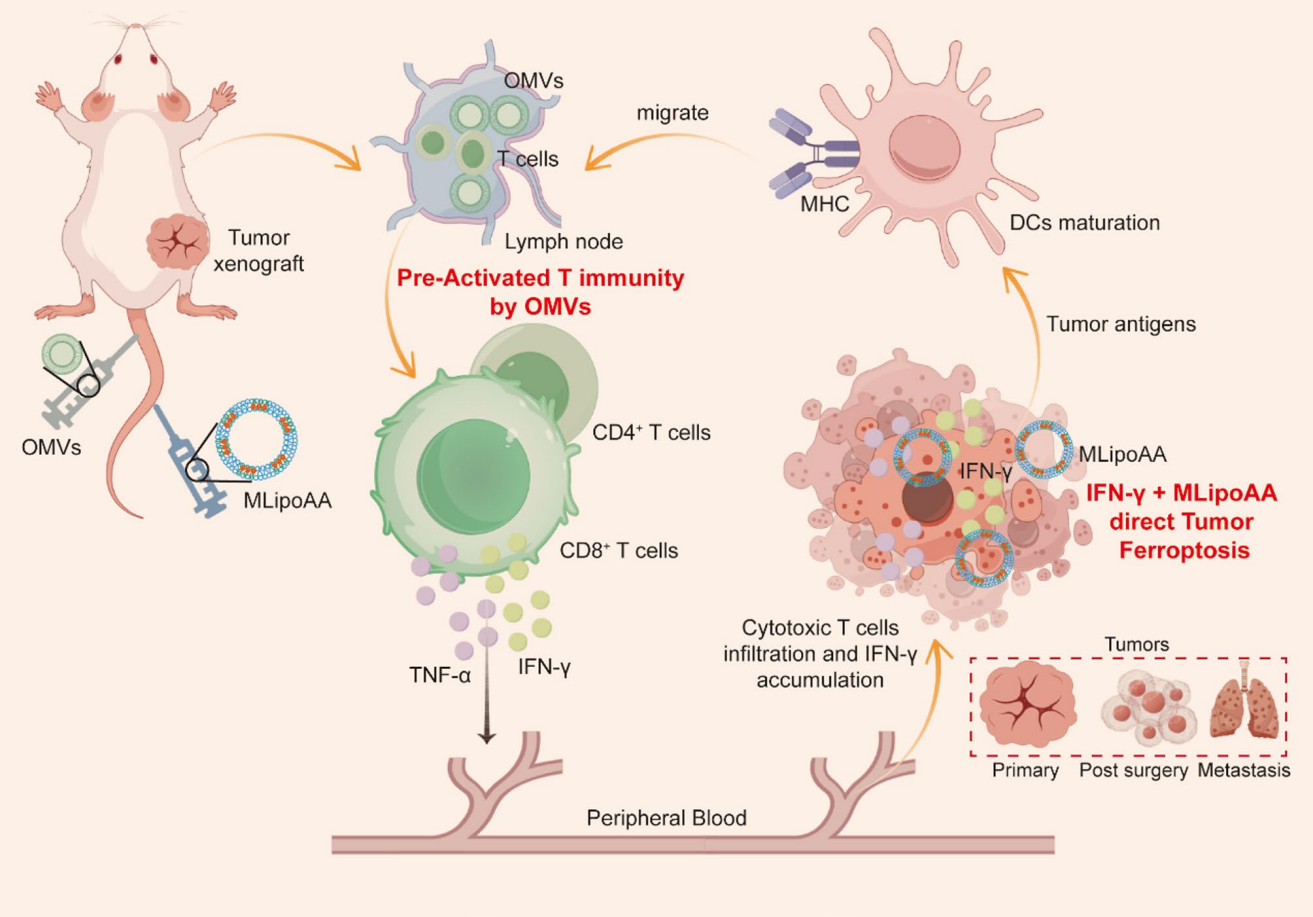
Schematic illustration of the dual-activation strategy using OMVs for T cell immunity pre-activation, producing IFN-γ. Administration of MLipoAA can be used to direct tumor-targeted IFN-γ-activated ferroptosis for effective tumor destruction and anti-metastasis

## Results and discussion

### Pre-activation of T cell immunity with engineered cytolysin containing OMVs

**Fig. 2:**
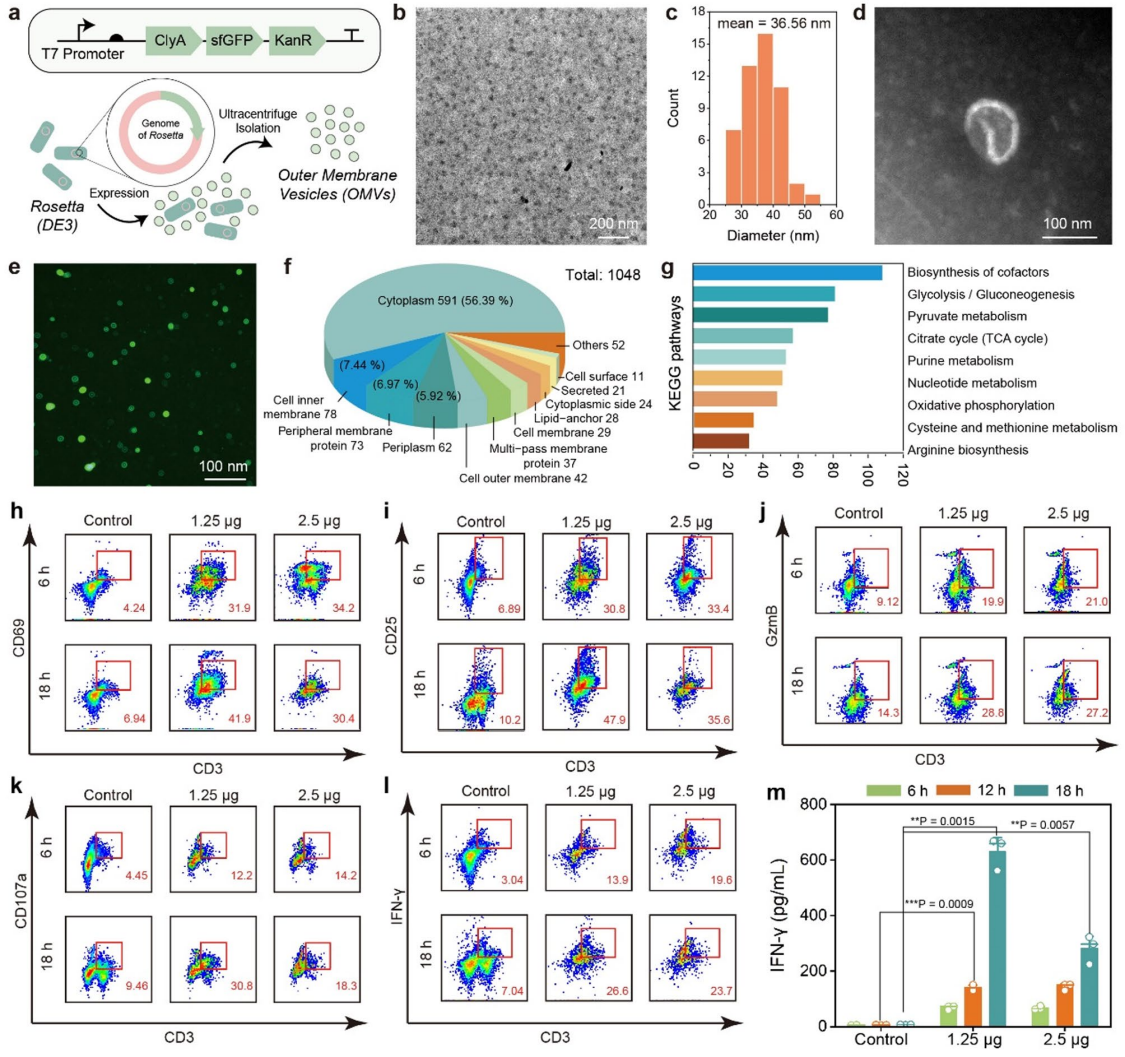
Activation of T lymphocytes by engineered OMVs. (**a**) Schematic illustration of the construction of plasmid, strain and OMVs isolation. (**b-d**) Representative bio-TEM images and diameter distributions of the isolated OMVs. (**e**) Fluorescence microscopic image of isolated OMVs. (**f**) Protein location analysis and (**g**) functional KEGG enrichment analysis of isolated OMVs. (**h-l**) Flow cytometry analysis of the percentage of (**h**) CD69, (**i**) CD25, (**j**) GzmB, (**k**) CD107a, and (**l**) IFN-γ gated on CD3 + T cells after co-incubation with OMVs at varied concentrations (0, 1.25 and 2.5 µg) for 6–18 h respectively. (**m**) Quantitative production of IFN-γ by T cells after OMVs stimulation at varied concentrations (0, 1.25, and 2.5 µg) for 6 h, 12–18 h respectively *via* ELISA

Bacterial derived OMVs are potent immune training agonists for immunopotentiation of both innate and adaptive immunities inside the tumor region. Cytolysin A (ClyA), being a pore-forming toxin, has been identified as effective tool for antigen display in vaccine application when secreted by OMVs. To obtain robust immune pre-activation performance of OMVs, we have genetically engineered *Escherichia coli Rosetta* (DE3) with recombinant plasmid pET28a carrying cytolysin A encoding gene ClyA and green fluorescent protein indicator (GFP) (Fig. 2a, Figure S1 in the Supporting Information for complete sequence). Western blot analysis of the strain confirms the expression of 6His-ClyA-sfGFP sequence (64.12 kDa) (Figure S2 in the Supporting Information). The genetically engineered strain was incubated at 20 °C and screened by GFP fluorescence on kanamycin-containing LB agar plates (Figure S3). Selected colonies were allowed to grow overnight in LB medium, followed by low-speed centrifugation of bacteria with GFP fluorescence and ultracentrifugation of supernatants with a filter membrane of 100 kDa cutoff to obtain concentrated OMVs (Figure S4). In order to compare OMVs from this genetically engineered E. coli with wild type E. coli (WT-OMVs), we collected OMVs and WT-OMVs simultaneously from 100 mL medium with 10^10^ cells, which results in 1.05 mg and 0.35 mg proteins separately as quantified by BCA assays (Figure S5). Besides, T cell immune activation effect of OMVs and WT-OMVs was also conducted, in which T cells were incubated with OMVs and WT-OMVs at equal concentration. The results indicate that OMVs exhibit better T cell activation effect especially under low doses (Figure S6). Therefore, clyA expression might induced higher vehicle excretion and pathogen immunity and OMVs obtained from this engineered E. coli was used for further research.

Bio-transmission electronic microscopy (Bio-TEM) images of OMVs feature with uniform bilayer vesicles of 36.56 nm on average in diameter (Fig. 2b-d). Under the confocal microscope, the OMVs appeared as small vesicles with constitutive expression of green fluorescence, corroborating the successful isolation of OMVs (Fig. 2e). With dynamic light scattering method, the hydrodynamic diameter and zeta potential of OMVs were measured to be 33.3 ± 5.3 nm and − 8.7 ± 3.1 mV respectively (Figure S7). Coomassie’s brilliant blue staining highlighted varied bands of bacteria-derived proteins found in OMVs (Figure S8). We then perform proteomics for components and functional analysis of OMVs. The protein constituents of OMVs majorly derive from the cytoplasm (56.39%), cell inner membrane (7.44%), peripheral membrane (6.97%), and periplasm (5.92%) (Fig. 2f), with their functionalities enriched to KEGG pathways such as biosynthesis of cofactors, glycolysis and purine metabolism, providing functional fundamentals to potentially regulate both innate and adaptive immunities (Fig. 2g).

We then evaluate the biosafety and immune activation performance of OMVs against T cells isolated from spleen lymphocytes. Negligible cytotoxicity at OMV dose of 1.25 µg (protein dose of OMVs) was observed. Higher concentration of OMVs (2.5 µg) led to a 22.9 ± 2.2% of T cell toxicity after 24 h co-incubation (Figure S9). We then evaluate the OMVs-initiated immune activation by quantification of CD69 + or CD25+ (activation markers for T cells) of CD3 + TILs with co-incubation period of 6 h and 18 h respectively. We found dose-dependent T-cell activation consequence by OMVs within 6 h. Statistically, OMVs at 1.25 µg induced 31.9% CD69^+^ and 30.8% CD25^+^ of CD3^+^ T lymphocytes, while untreated T cells exhibit only 4.2% and 6.9% of the T cells respectively (Fig. 2h-i). The expressions of these markers further increased (34.2% for CD69 + T cells and 33.4% for CD25 + T cells) when the dose of OMVs reached 2.5 µg for a co-incubation period of 6 h. Elongation of the co-incubation time to 18 h led to an enhanced activation of T cells (41.9% for CD69^+^ T cells and 47.9% for CD25 + T cells) at a low dose of OMVs (1.25 µg). Nevertheless, the expression populations of CD69^+^ and CD25^+^ T cells were decreased to 30.4% and 35.6% respectively, possibly due to the cytotoxic effect of OMVs against T cells. Effector markers of TILs such as GzmB, CD107a, and IFN-γ have also been analyzed. Following similar trending to CD69 and CD25 expressions, OMVs induce dose-dependent expression of GzmB, CD107a, and IFN-γ in 6 h co-incubation. In 18 h co-incubation with OMVs (1.25 µg), the cell populations for GzmB + and CD107a + T cells were quantified to be 28.8% and 30.8% respectively (Fig. 2j-k). Corresponding statistical analysis exhibits that optimal conditions for activation and effector induction of CTLs are OMVs co-incubation with low dose (1.25 µg) and elongated time (18 h) (Figure S10). We also found that IFN-γ + CTLs have been quantified to be maximally 26.6% at optimal stimulation conditions *via* flow cytometry (Fig. 2l). Quantitative enzyme-linked immunosorbent assay (ELISA) of CTLs supernatants after OMVs stimulation at different conditions indicate a secretion of IFN-γ as high as 6.67 ng/mL after a coincubation period of 18 h with 1.25 µg OMVs (Fig. 2m). Under the same conditions, we also measured TNF-α secretion by stimulated CTLs to be 60 pg/mL, the highest concentration among all evaluation conditions (Figure S11). Therefore, we found that the activation of T cells by OMVs can be significantly affected by the cell cytotoxicity of OMVs against the primary T cells temporally. Although high dose of OMVs (2.5 µg) may induce cytotoxicity to the T cells, rational dosage of OMVs (1.25 µg) can effectively stimulate optimal activation of T cell immunity through co-incubation period of 18 h, producing abundant cytokines (i.e., IFN-γ and TNF-α) for potential antitumor responses.

### Engineered OMVs direct arachidonic acid-induced tumor ferroptosis

**Fig. 3:**
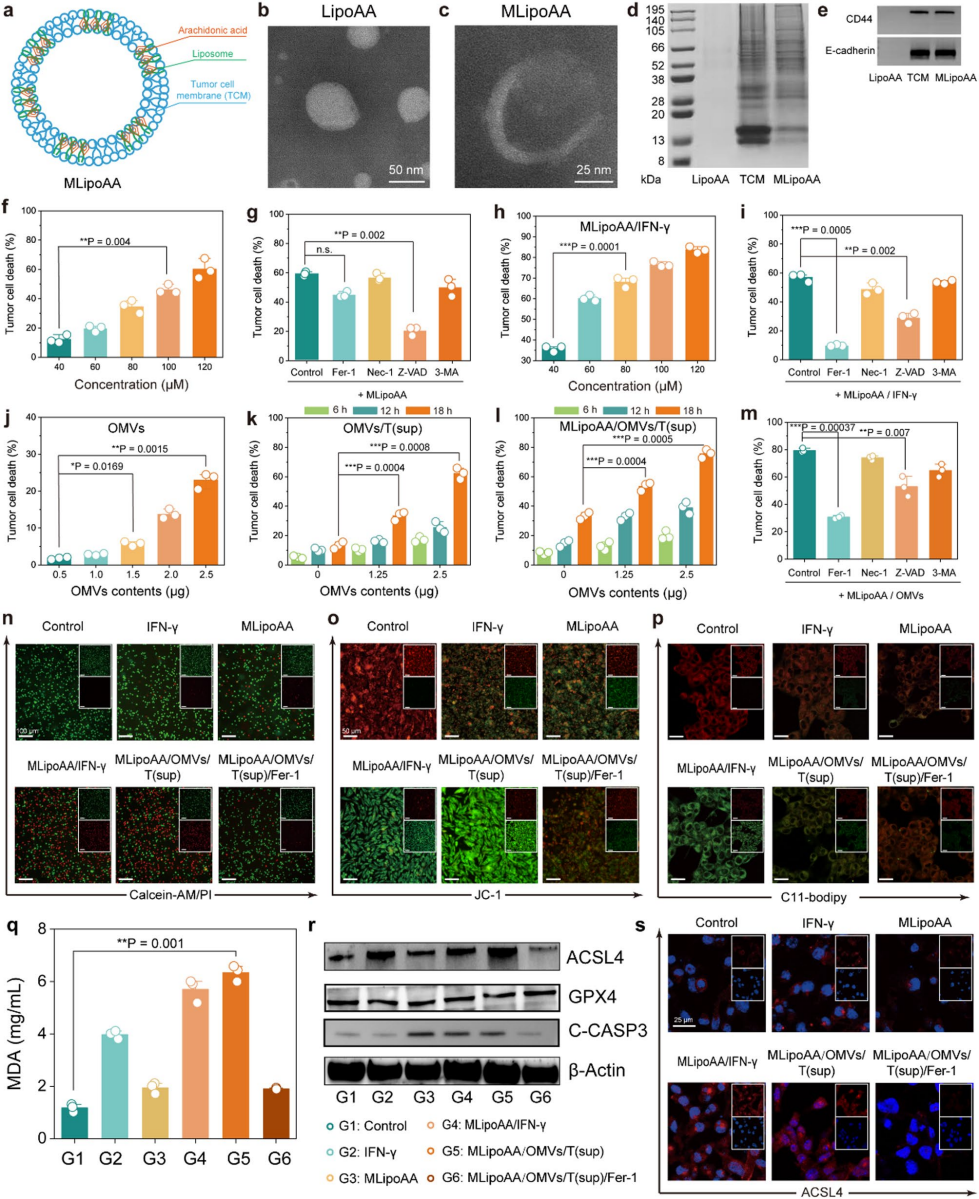
In vitro cellular experiments of ferroptosis induction by the combination of OMVs-activated T cells and MLipoAA. (**a**) Schematic illustration of MLipoAA. TEM images of (**b**) LipoAA and (**c**) MLipoAA. (**d**) Coomassie brilliant blue staining and (**e**) western blot analysis of CD44 and E-cadherin expression of LipoAA, TCM, and MLipoAA. (**f**) Cytotoxicity of MLipoAA against CT26 tumor cells at varied concentrations (40, 60, 80, 100, and 120 µM). (**g**) CT26 tumor cell death of MLipoAA at 120 µM after the addition of Fer-1, Z-VAD, Nec-1, or 3-MA. (**h**) Cytotoxicity of MLipoAA against CT26 tumor cells at varied concentrations (40, 60, 80, 100, and 120 µM) in combination with IFN-γ at 10 ng/mL and (**i**) corresponding cell death rescue after the addition of Fer-1, Z-VAD, Nec-1 or 3-MA respectively. (**j**) CT26 tumor cell death of OMVs at varied concentrations (0.5, 1.0, 1.25, 2.0, and 2.5 µg). (**k**) CT26 tumor cell death of T cell supernatants activated by OMVs at 0, 1.25, or 2.5 µg for 6 h, 12 h, and 18 h, and (**l**) corresponding cell death in combination with 60 µM MLipoAA. (**m**) CT26 tumor cell death induced by 60 µM MLipoAA in combination with 2.5 µg OMVs-activated T cell supernatants after the addition of Fer-1, Z-VAD, Nec-1, or 3-MA. Co-incubation time of 18 h. (**f-m**) *n* = 3, biological replicates. Data are presented as they are and mean ± s.d. (**n-p**) Confocal microscopic images of CT26 cells after co-incubation with medium containing IFN-γ (10 ng/mL), MLipoAA (60 µM), MLipoAA (60 µM) in combination with IFN-γ (10 ng/mL), MLipoAA in combination with OMVs (1.25 µg) stimulated T cell supernatants (t = 18 h), MLipoAA in combination with OMVs (1.25 µg) stimulated T cell supernatants (t = 18 h) supplemented with Fer-1 (2 µM), followed by staining with (n) calcein-AM/PI (scale bar: 100 μm), (**o**) JC-1 (scale bar: 50 μm) or (**p**) C11-bodipy (scale bar: 25 μm). (**q**) MDA assays of CT26 cells after co-incubation with medium containing IFN-γ (10 ng/mL), MLipoAA (60 µM), MLipoAA (60 µM) in combination with IFN-γ (10 ng/mL), MLipoAA in combination with OMVs (1.25 µg) stimulated T cell supernatants (t = 18 h), MLipoAA in combination with OMVs (1.25 µg) stimulated T cell supernatants (t = 18 h) supplemented with Fer-1 (2 µM). *n* = 3, biological replicates. Data are presented as they are and mean ± s.d. (**r**) Western blot of Acsl4, Gpx4 and cleaved caspase-3 quantification of CT26 cells after co-incubation with medium containing IFN-γ (10 ng/mL), MLipoAA (60 µM), MLipoAA (60 µM) in combination with IFN-γ (10 ng/mL), MLipoAA in combination with OMVs (1.25 µg) stimulated T cell supernatants (t = 18 h), MLipoAA in combination with OMVs (1.25 µg) stimulated T cell supernatants (t = 18 h) supplemented with Fer-1 (2 µM). (**s**) Immunofluorescence microscopic images of Acsl4-stained CT26 cells after co-incubation with medium containing IFN-γ (10 ng/mL), MLipoAA (60 µM), MLipoAA (60 µM) in combination with IFN-γ (10 ng/mL), MLipoAA in combination with OMVs (1.25 µg) stimulated T cell supernatants (t = 18 h), MLipoAA in combination with OMVs (1.25 µg) stimulated T cell supernatants (t = 18 h) supplemented with Fer-1 (2 µM). Scale bar: 25 μm

Arachidonic acid (AA) is a polyunsaturated fatty acid derived from the hydrolysis of phospholipid. Accumulation of AA is a potential trigger of cell ferroptosis. To selectively induce tumor ferroptosis, AA was hybridized and stabilized in the hydrophobic layer of liposome (LipoAA), with further co-extrusion with CT26 tumor cell membrane mechanically, forming MLipoAA for further in vitro and in vivo experiments (Fig. 3a). The as-synthesized LipoAA exhibits a typical vesicular morphology with a mean particle size of 110 nm as confirmed by TEM (Fig. 3b). An evident membrane structure with a larger particle size of MLipoAA was also revealed (Fig. 3c). Dynamic light scattering measurements reveal the uniform hydrodynamic diameter distribution around 145.3 nm of LipoAA and 166.8 nm of MLipoAA, while zeta potentials of LipoAA before (−22.3 ± 1.1 mV) and after tumor cell membrane coating (−20.5 ± 1.0 mV) were not significantly changed (Figure S12-13). Coomassie brilliant blue staining after SDS-PAGE indicates that MLipoAA contains major proteins originated from the CT26 tumor cells (Fig. 3d). Western blot analysis further identified the specific markers from the membrane including CD44 and E-cadherin (typical surface proteins associated with cell recognition and tight junction respectively) (Fig. 3e). The loading efficiency of AA in MLipoAA was quantified to be 0.04 mg/µL using high-performance liquid chromatography after demulsification of the nanoliposome with ethanol (Figure S14). MLipoAA was further stained with lipophilic, near-infrared fluorescent cyanine dye DiR dye for cellular internalization evaluation. In a time-dependent manner, MLipoAA could be effectively endocytosized inside the tumor cells in 2 h. Accumulation of MLipoAA inside tumor cells for 8 h could also be observed by CLSM (Figure S15). Considering that arachidonates can be protonated under low pH environment, leading to the formation of hexagonal crystals and subsequent drug release from the nanoliposome. At pH = 5.0, MLipoAA exhibits a larger particle size and decreased zeta potential, implicating the structural destruction (Figure S16) and accelerated release of arachidonic acid (Figure S17-S18).

Both IFN-γ and AA exhibited dose-dependent cytotoxicity against CT26 cells. Specifically, 50 ng/mL of IFN-γ induced 23.3% of cell death (Figure S19), while 120 µM of MLipoAA could effectively kill 60.5% of the cells in 24 h co-incubation (Fig. 3f). To evaluate the cell death process triggered by IFN-γ or MLipoAA, various cell death pathway inhibitors including Ferrostatin-1 (Fer-1, a ferroptosis inhibitor), carbobenzoxy-valyl-alanyl-aspartyl-[O-methyl]-fluoromethylketone ketone (Z-VAD, a caspase inhibitor to inhibit apoptosis), necrostatin-1 (Nec-1, a necroptosis inhibitor) and 3-Methyladenine (3-MA, an autophagy inhibitor) were supplemented to CT26 tumor cells treated with IFN-γ (10 ng/mL) or MLipoAA ([AA] = 120 µM). Only when supplementation of Z-VAD could reduce the cell death percentage from 61.6% to 22.7%, indicating that MLipoAA mainly kills tumor cells through apoptosis (Fig. 3g). The combination of MLipoAA ([AA] = 60 µM) and IFN-γ (10 ng/mL) significantly enhanced cytotoxicity against CT26 cells, resulting in 61.8% cell death (Fig. 3h). Mechanistic studies revealed that Fer-1 and Z-VAD reversed 47% and 27.9% of this cell death respectively (Fig. 3i). Notably, upon the addition of IFN-γ, the proportion of cell death rescued by Z-VAD decreased from 38.9% (MLipoAA alone) to 27.9%, whereas that rescued by Fer-1 increased from 14% to 47%. These results indicate that the combination treatment shifts the primary mode of cell death from apoptosis to ferroptosis, with ferroptosis becoming the dominant mechanism.

Since OMVs could stimulate the CTLs to release IFN-γ, the supernatants of CTLs after OMVs stimulation at different co-incubation conditions was employed to treat CT26 tumor cells instead of the co-incubation of free IFN-γ. In a dose dependent manner, the engineered OMVs can kill 5.5% and 22.3% of CT26 tumor cells at doses of 1.25 µg and 2.5 µg respectively (Fig. 3j). To indicate the consequence of T cells after OMVs stimulation, the supernatants of CTLs after OMVs stimulation (OMVs/T(sup), containing cytokines such as IFN-γ) was collected for cytotoxicity analyses. For instance, the supernatant derived from the stimulated CTLs by OMVs (1.25 µg, co-incubation time: 18 h) induce 30.2% of cell death, demonstrating the prominent cell-killing effect of the cytokines from OMVs-activated CTLs (Fig. 3k). It is interesting that even though cell viability of CTLs decreased under 2.5 µg OMVs stimulation for 18 h, the derived supernatant mediated cytotoxicity against CT26 cells was still enhanced, possibly due to the premature release of potent cytokines ahead of the cell death of the CTLs. The cytotoxicity was further enhanced by the addition of 60 µM MLipoAA (Fig. 3l). In the OMVs/T(sup)/MLipoAA combination group, which induced 79.38% cell death, Fer-1 and Z-VAD rescued 48.66% and 26.48% of the cell death, respectively (Fig. 3m). These results suggest that ferroptosis contributes substantially to the tumor cell death induced by the combination of MLipoAA and cytokines secreted from OMVs-activated CTLs.

The cytotoxicity effect was further visualized by cell staining of Calcein-AM and propidium iodide (PI). Cells with different treatments were allocated into different groups respectively: Control (untreated); IFN-γ (10 ng/mL); MLipoAA (60 µM); IFN-γ + MLipoAA (10 ng/mL IFN-γ + 60 µM MLipoAA); OMVs/T(sup)/MLipoAA (supernatant of OMVs-stimulated CTLs + MLipoAA 60 µM) and OMVs/T(sup)/MLipoAA/Fer-1 (supernatant of OMVs-stimulated CTLs + MLipoAA 60 µM + Fer-1). A major population of cells from the OMVs/T(sup)/MLipoAA group were stained with red fluorescence as compared to the other groups, implicating the most robust cell-killing effect. We also found that a majority of the cells from the OMVs/T(sup)/MLipoAA/Fer-1 group survived under Fer-1 co-incubation, further confirming the ferroptotic cell death enabled by OMVs/T(sup)/MLipoAA (Fig. 3n).

Mitochondrial damage is a typical pathological feature during ferroptotic cell death. Bio-TEM images of cells from the OMVs/T(sup)/MLipoAA group reveal a ferroptotic phenotype in the mitochondria, as characterized by a condensed mitochondrial structure with markedly reduced mitochondrial ridges and a smaller mitochondrial volume (Figure S20). As a mitochondrial damage indicator, JC-1 probe could transform from red fluorescent J-aggregates to cationic JC-1 dye with green fluorescence upon the loss of the mitochondrial electrochemical potential of the cells with OMVs/T(sup)/MLipoAA treatment (Fig. 3o). Intracellular lipid peroxidation was further verified by the C11-BODIPY probe (Ex/Em = 581/591 nm). Notably, a substantial shift towards higher oxidation conditions was observed in the cells from the OMVs/T(sup)/MLipoAA group by flow cytometry, providing evidence of the enhanced lipid peroxidation (Figure S21). It was also observed in fluorescence microscopy that cells from both the IFN-γ/MLipoAA group and OMVs/T(sup)/MLipoAA group induce strong green fluorescence, originating from the oxidized C11-BODIPY. Such fluorescence could be further diminished after Fer-1 co-incubation (Fig. 3p). In addition, the end-product of lipid peroxides malondialdehyde (MDA), was also significantly elevated by IFN-γ/MLipoAA and OMVs/T(sup)/MLipoAA, confirming the ferroptosis cell fate (Fig. 3q). The expressions of two key ferroptosis mediators, Acsl4 (Acyl-CoA synthetase long-chain family member 4) and Gpx4 (Glutathione peroxidase 4) were further evaluated by western blot. The quantification of the Acsl4 blot from the OMVs/T(sup)/MLipoAA was 2.24 folds compared to the blot from the control group. With Fer-1 treatment, the expression of the blot was decreased. On the other hand, the Gpx4 blots did not show significant variations between groups, validating that OMVs/T(sup)/MLipoAA treatment induces ferroptosis in CT26 tumor cells *via* Acsl4 rather than Gpx4. Furthermore, consistent with our previous conclusion that apoptosis is the major cell death mechanism in the MLipoAA group while ferroptosis dominates in the MLipoAA/IFN-γ and MLipoAA/OMVs/T(sup) groups, we observed a corresponding decrease in cleaved caspase-3 expression in the latter two groups (Fig. 3r). From confocal microscopic images, we found that CT26 tumor cells incubated with IFN-γ/MLipoAA exhibited brighter red fluorescence signals along cell membrane, indicating a higher expression of Acsl4 intracellularly as compared to untreated cells or cells from other groups (Fig. 3s). Collectively, we validated that the engineered OMVs could effectively produce IFN-γ, directing arachidonic acid to trigger tumor ferroptosis through Acsl4 mediator.

### Preactivated immunity by OMVs directs in vivo ferroptotic therapeutics in combination with MLipoAA

**Fig. 4:**
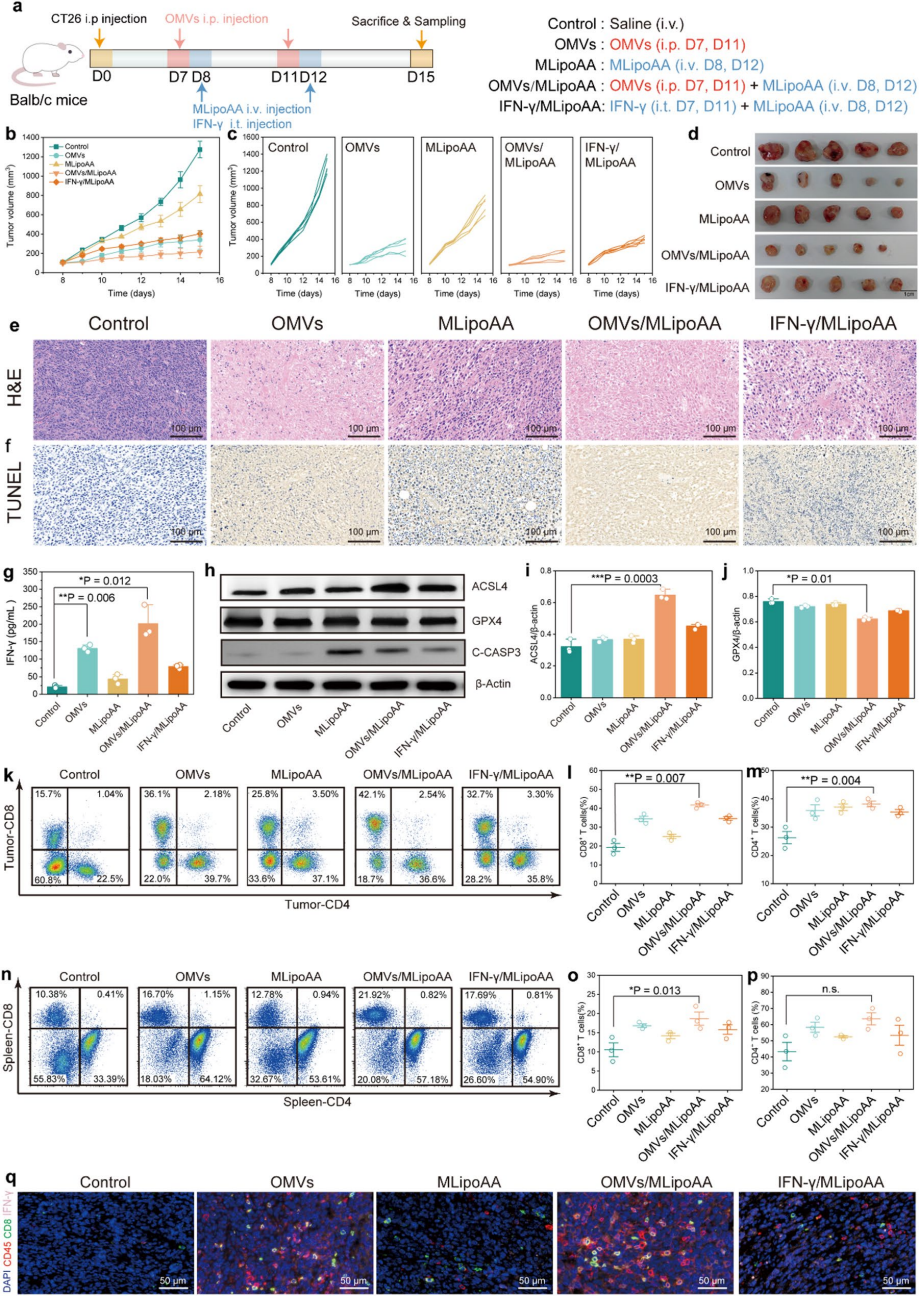
In vivo experiments by combination therapeutics of OMVs and MLipoAA. (**a**) Therapeutic schedule of in vivo experiments by combination therapeutics of OMVs and MLipoAA on CT26-tumor bearing Balb/c mice. (**b-c**) Tumor growth curves of mice from control, OMVs, MLipoAA, OMVs/MLipoAA, and IFN-γ/MLipoAA groups. (**d**) Digital photographs of dissected tumor xenografts at the end of the experiment. (**b-d**) *n* = 5, biological replicates. Data are presented as mean ± s.d. (**e-f**) Histopathological images of tumor tissues of mice from each group stained with (**e**) H&E and (**f**) TUNEL. (**g**) Serum IFN-γ concentration evaluation at day 15 after combinational administrations. (**h-j**) WB evaluations (**i**) and corresponding statistical analysis (**l-j**) of ACSL4, GPX4 and cleaved caspase-3 proteins in tumor tissues of mice from different groups. (**k-m**) Representative flow cytometry profile (**k**) and corresponding statistical analysis (**l-m**) of CD4 + and CD8 + tumor-infiltrating lymphocyte. (**n-p**) Representative flow cytometry profile (**o**) and corresponding statistical analysis (**o-p**) of CD4 + and CD8 + T cells within spleen tissues. (**g-j**) *n* = 3, biological replicates. Data are presented as they are and mean ± s.d. (**q**) Representative polychromatic immunofluorescent staining images of tumor sections of mice from different groups: Panel information: CD45+ (red), CD8+ (green), IFN-γ (pink), and DAPI (blue)

To evaluate the in vivo therapeutic consequences of preactivated immunity by OMVs in combination with MLipoAA, twenty-five Balb/c mice were randomly allocated into five groups (Control, OMVs, MLipoAA, OMVs/MLipoAA, and IFN-γ/MLipoAA) prior to the establishment of murine subcutaneous CT26 xenograft models. OMVs (250 ng/g) were intraperitoneally injected 24 h before intravenous administration of MLipoAA (2 mg/kg) to stimulate host immunity. IFN-γ was intratumorally injected 24 h before MLipoAA injection to induce tumor ferroptosis without pre-activation of the host immunity (Fig. 4a). The body weight of mice in each group exhibits no significant variances during the evaluation period of 15 days (Figure S22). Mice from the control group exhibit aggressive and exponential tumor growth. Treatment with MLipoAA monotherapy suppressed the growth of tumor xenografts with an inhibition rate of 36.1%. When combined with IFN-γ, the inhibition rate increased to 58.3%, suggesting the potential involvement of tumor ferroptosis. Notably, OMVs treatment alone achieved an inhibition rate of 63.3%. The most significant result was achieved by the OMVs/MLipoAA combination, which suppressed tumor growth by 81.6%, underscoring its superior therapeutic profile (Fig. 4b-d). The antitumor effect of OMVs is driven by sustained IFN-γ production and long-lasting immune responses [24], which appear to synergistically enhance tumor ferroptosis in combination with MLipoAA.

Further evaluation through hematoxylin and eosin (H&E) and terminal deoxynucleotidyl transferase-mediated dUTP nick end-labeling (TUNEL) staining of the tumor sections identified that fewer cell populations and more prominent tumor cell death could be observed when the mice were treated with OMVs/MLipoAA, as compared to the control group (Fig. 4e-f). Besides, H&E staining of the heart, liver, spleen, lungs, and kidneys reveals no discernible pathological damages (Figure S23). The complete blood routine and blood biochemistry analyses suggest that no significant adverse effect was generated from OMVs/MLipoAA therapeutics (Figure S24). To explore the tumor cell fate induced by OMVs/MLipoAA in vivo, the serum IFN-γ level, as a coordinator to trigger Acsl-dependent ferroptosis, was detected using ELISA. The outcome indicates an increase from 25 pg/mL in the control group to 130 pg/mL in the OMVs group and 178 pg/mL in the OMVs/MLipoAA group on day 15, validating that IFN-γ is continuously produced by OMVs-preactivated CTLs (Fig. 4g). Moreover, WB analysis displays a 32% increase in ACSL4 expression in the OMVs/MLipoAA group and a slight decrease in GPX4 expression, as compared to the control group, indicating a robust stimulation of the ACSL4-mediated tumor ferroptosis in vivo (Fig. 4h-j). Furthermore, cleaved caspase-3 expression in OMVs/MLipoAA group and IFN-γ/MLipoAA group were both declined comparing with MLipoAA group (Figure S25). While the combination treatment does enhance ferroptosis (elevated ACSL4 and declined cleaved capase-3 expression), the exact extent of ferroptosis contribution to the overall therapeutic effect in vivo still warrants further investigation and clarification.

**Fig. 5:**
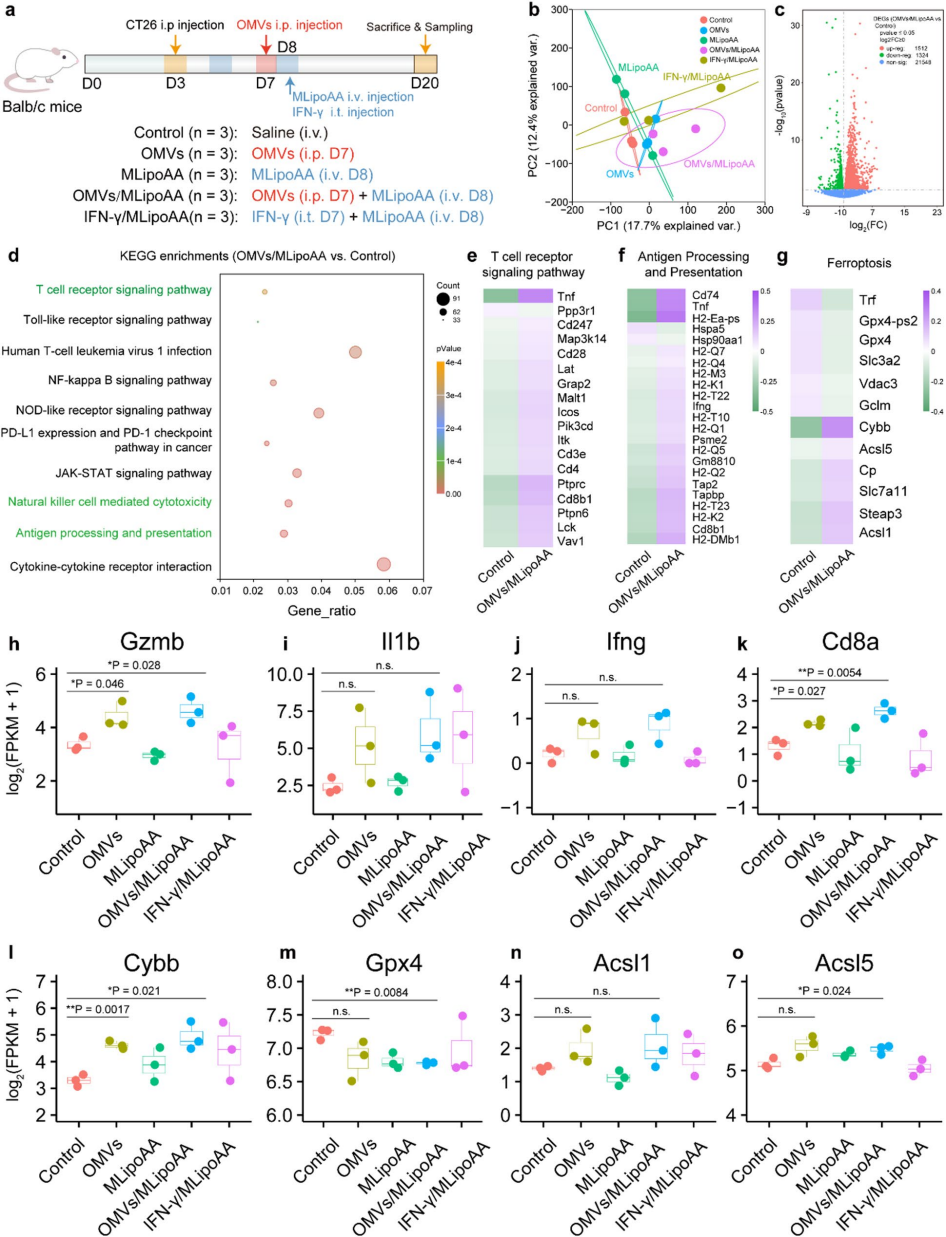
Transcriptional Analysis of OMVs Pre-activation Triggered Ferroptotic Therapeutics. (**a**) Schematic schedule of in vivo ferroptotic therapy for transcriptional analysis. (**b**) PCA plot of tumor samples of mice from different groups. (**c**) Volcano plot of DEGs between OMVs/MLipoAA group and control group. (**d**) KEGG analysis of DEGs between OMVs/MLipoAA group and control group. Heatmap of gene distributions in (**e**) T cell receptor signaling, (**f**) antigen processing and presentation, and (**g**) ferroptosis pathways. mRNA expressions of anti-tumor immunity-associated genes including (**h**) Gzmb, (**i**) Il1b, (**j**) Ifng, (**k**) Cd8a, and ferroptosis-associated genes including (**l**) Cybb, (**m**) Gpx4, (**n**) Acsl1 and (**o**) Acsl5. (**b-o**) *n* = 3, biological replicates. Data are presented as they are and mean ± s.d

OMVs mediated immunopotentiation was evaluated *via* stiffness-based elastography. An intraperitoneal injection of OMVs resulted in a significant increase in tumor stiffness, implicating the local infiltration of inflammation (Figure S26). The maturation of dendritic cells (DCs) plays key roles in antigen presentation process to trigger adaptive immunity. The percentage of CD11c + CD80 + CD86 + DCs increased specifically from 16.9% in the Control group to 44.3% in the OMVs/MLipoAA group, even higher than that of the OMVs group (31.2%) (Figure S27). These results show that this combinational ferroptotic induction strategy trigger potent immunogenic cell death for DCs maturation. The representative flow cytometry results of TILs exhibit gradually increased of CD8 + T cells from 15.7% (control) to 42.1% (OMVs/MLipoAA) (Fig. 4k-m). The increase was also observed for the CD8 + T cells analyzed from spleen tissue from 10.38% (control) to 21.92% (OMVs/MLipoAA) (Figure S28, Fig. 4o-q). Flow cytometry and statistical analysis of CD86 + CD206- tumor associated macrophages (TAMs) also validate the elevated M1 phenotype macrophages for tumor inhibition (Figure S29). Additionally, immunofluorescence analysis of tumor tissues reveals a substantial increase of CD45+, CD8 + T cells. IFN-γ infiltration was observed in the tumor section from OMVs/MLipoAA group, suggesting effective and long-lasting immunopotentiation effect of OMVs/MLipoAA (Fig. 5r). Collectively, preactivated immunity by OMVs directs in vivo ferroptotic therapeutics in combination with MLipoAA could be validated.

### Transcriptional analysis of OMVs pre-activation potentiated MLipoAA-enabled ferroptotic therapeutics

 To further systematically investigate the biological effects of OMVs pre-activation induced ferroptotic tumor therapy in synergy with MLipoAA in vivo, we conducted mRNA transcriptional analysis of tumor tissues 12 days post treatment, to validate the occurrence of ferroptosis and the subsequent activation of both innate and adaptive immune responses (Fig. 5a). The PCA graph reveals significant differences between the Control group and OMVs/MLipoAA group (Fig. 5b), indicating substantial transcriptional variations. Defferentially expressed genes (DEGs) between control and OMVs/MLipoAA groups have been depicted in the volcano plot for pairwise comparison, in which 1,512 genes were upregulated and 1,324 genes were down-regulated (Fig. 5c). These genes are found to enrich to KEGG pathways including T cell receptor signaling pathway, natural killer cell-mediated cytotoxicity, and antigen processing and presentation. These findings suggest long-term antitumor immunity for as long as 13 days post OMVs administration (Fig. 5d). From T cell receptor signaling pathway, Tnf, Cd28, Lat, Cd3e, Cd4, and Cd8b1 were found with significant upregulation after OMVs/MLipoAA treatment (Fig. 5e). Likewise, for antigen processing and presentation pathway, associated genes such as Cd74, Ifng, and H2-Q5 were significantly upregulated (Fig. 5f). Although ferroptosis pathway was not significantly enriched (p-value of 0.2013), ferroptosis-related genes, such as Trf, Gpx4, and Slc3a2 were significantly down-regulated, and Cybb, Acsl5, Slc7a11, and Acsl1 were significantly upregulated (Fig. 5g). To validate the ferroptosis potentiated by OMVs pre-activated immunity, immunity-associated genes such as Gzmb, Il1b, Ifng, and Cd8a were also quantified as normalized expressions for all the groups. As we observed, both tumors from OMVs and OMVs/MLipoAA groups induced significant upregulation of these antitumor immunity markers, validating the immunopotentiation effect of OMVs pre-activated immunity. IFN-γ/MLipoAA group exhibited lower expression result, mainly due to the insufficient bioavailability of the IFN-γ cytokine to trigger constant immunity at tumor site (Fig. 5h-k). Furthermore, OMVs/MLipoAA group exhibited the most significant changes in the expressions of ferroptosis-related genes such as Cybb, Gpx4, Acsl1, and Acsl5 (Fig. 5l-o). Further investigation is needed to clarify the mechanistic underpinnings of the combination therapy, particularly to define the central role of ferroptosis in tumor therapy.

### OMVs pre-activation directs MLipoAA-enabled ferroptotic therapy inhibits tumor recurrence

**Fig. 6:**
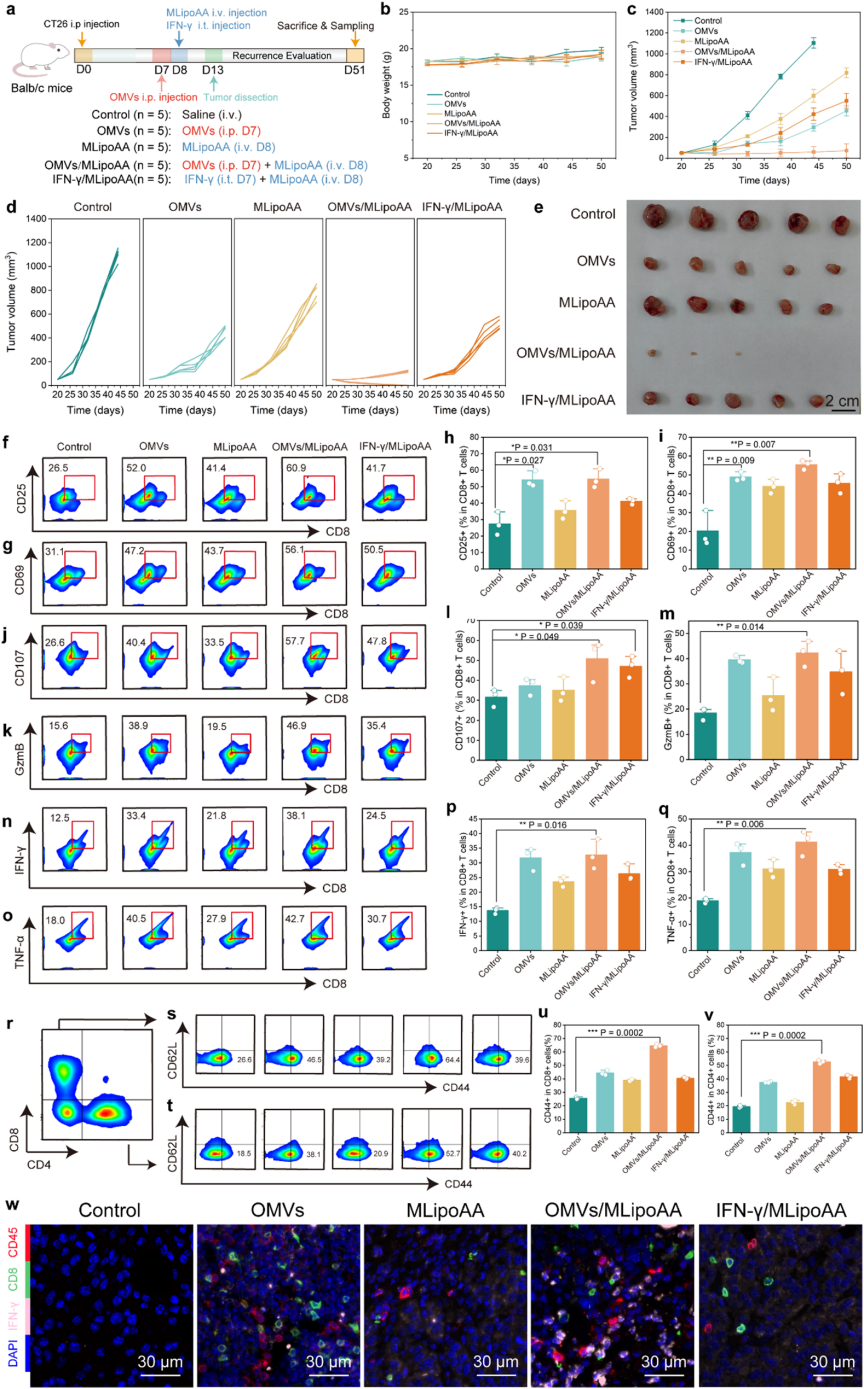
In vivo experiments of OMVs pre-activation directs MLipoAA-enabled ferroptotic therapy inhibits tumor recurrence. (**a**) Schematic schedule of the in vivo experiment evaluation. (**b**) Body weight of mice from different groups during 50-day observation. (**c-d**) Tumor growth curves of recurrent tumors of mice from different groups. (**e**) Digital photographs of dissected recurrent tumor tissues at the end of evaluation. *n* = 5, biological replicates. Data are presented as mean ± s.d. (**f-n**) Representative flow cytometry profiles of (**f**) CD8 + CD25+, (**g**) CD8 + CD69+, (**j**) CD8 + CD107a+. (**k**) CD8 + GzmB+, (**n**) CD8 + IFN-γ+, and (**o**) CD8 + TNF-α + T cells isolated from recurrent tumor tissues and (**h-q**) corresponding statistical analysis. (**r-v**), Representative flow cytometry profiles of CD44CD62L T cells in each group (**r-t**) and statistical analysis (**u-v**). (f-v) *n* = 3, biological replicates. Data are presented as they are and mean ± s.d. w, Representative polychromatic immunofluorescent staining images of tumor sections of mice from different groups. Panel: CD45+ (red), CD8+ (green), IFN-γ (pink), and DAPI (blue)

We next seek for therapeutic potentials against tumor recurrence. Twenty-five Balb/c mice were randomly allocated into five groups prior to the subcutaneous colon xenograft establishment. Mice were administered with OMVs or IFN-γ on Day 7, MLipoAA on Day 8. The tumor xenografts were surgically dissected with a remaining volume of 50 mm^3^ on Day 13 (Fig. 6a). No significant differences were observed on the body weight of mice from different groups (Fig. 6b). The tumor growth curves reveal the rapid recurrence of mice from control group, while suppressed tumor recurrence could be observed for mice from OMVs or MLipoAA groups. Notably, an almost 95.7% inhibition rate of tumor recurrence was reached by combinational therapeutics of OMVs/MLipoAA throughout the entire 50-day observation, highlighting its considerable potential in inhibiting malignant tumor recurrence (Fig. 6c-e).

The therapeutic outcome may be attributed to effective host immune activation directing tumor ferroptosis for prominent vaccination effect. Initially, T-cell immunity was evaluated by isolating the TILs from the recurrent tumor tissues. Flow cytometry indicates an increase of 25.5% of CD8 + CD25 + T cells and 16.1% increasement of CD8 + CD69 + T cells from OMVs group. These populations further increased to 60.9% and 56.1% in the cells from OMVs/MLipoAA group (Fig. 6f-i, Figure S30). The maximum CD8 + CD107a + T cells and CD8 + GzmB + T cells proportions were also observed in recurrent tumors after combinational OMVs/MLipoAA therapeutics, reaching 57.7% and 46.9% respectively (Fig. 6j-m). Besides, the populations of CD8 + IFN-γ + and CD8 + TNF-α + T cells from OMVs/MLipoAA group are quantified to be 38.1% and 42.7% respectively, ranking the highest expressions among control and other treatment groups (Fig. 6n-q). Both effector memory T cells (CD62L-CD44+) in CD8 + and CD4 + subsets exhibit statistically significant increase when tumors were treated with combinational OMVs/MLipoAA, reaching maximum population percentages of 64.4% and 52.7% respectively (Fig. 6r-v). Immunofluorescence analysis of recurrent tumor tissues further demonstrate enhanced CD45, CD8, and IFN-γ positive cells distributed inside tumor sections from OMV/MLipoAA group (Fig. 6w). From the above analyses, OMVs pre-activation directs MLipoAA-enabled ferroptotic therapy could further trigger vaccination effect, promoting long-term anti-tumor memory to combat tumor recurrence.

### OMVs pre-activation directs MLipoAA-enabled ferroptotic therapy inhibits tumor metastasis

**Fig. 7:**
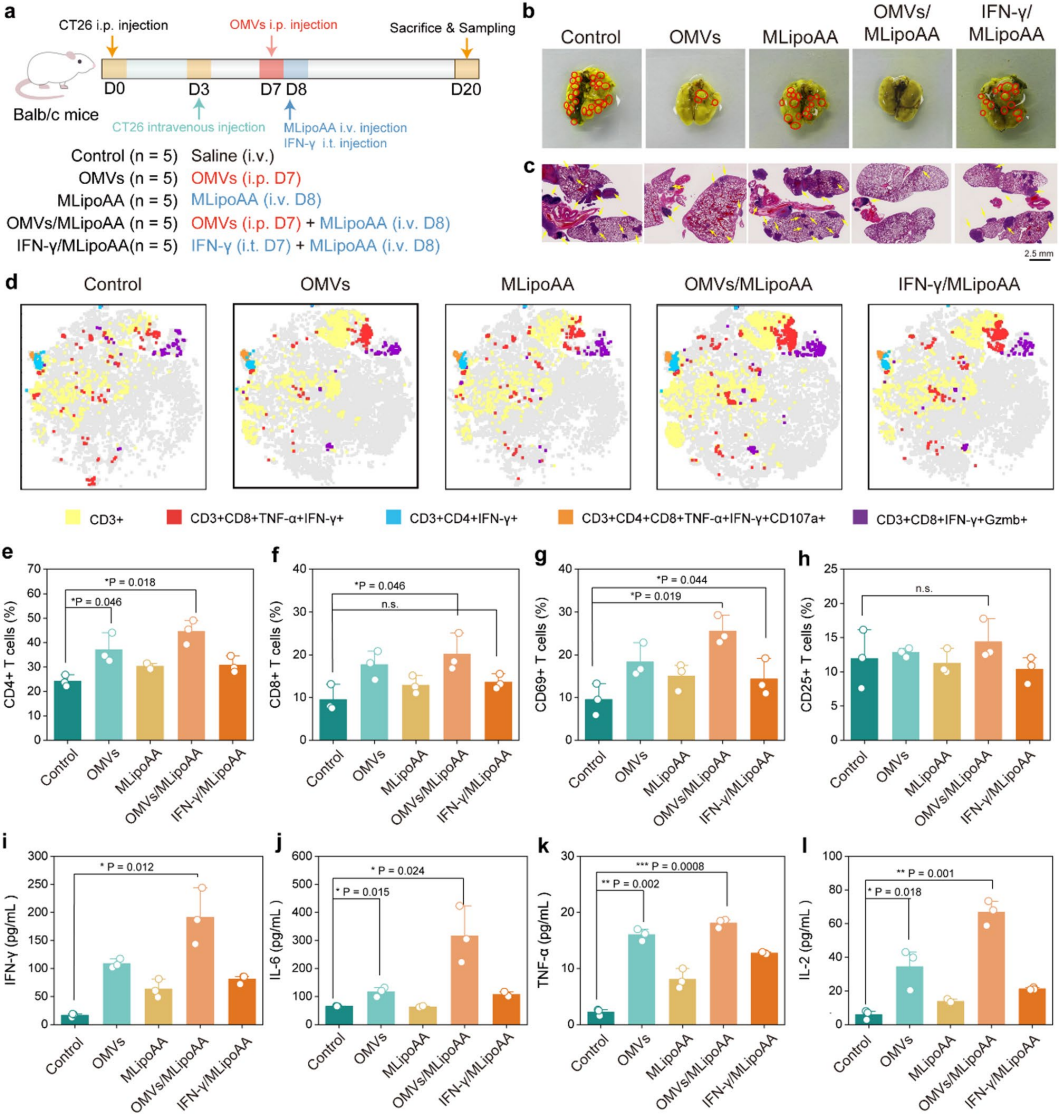
OMVs Pre-activation Directs MLipoAA-enabled Ferroptotic Therapy Inhibits Tumor Metastasis. (**a**) Schematic schedule of the in vivo anti-metastasis experiments on Balb/c mice. (**b**) Digital photographs of dissected lung tissues with Bouin’s fixation of mice from different treatment groups. (**c**) Microscopic images of H&E-stained lung tissues of mice from different treatment groups for tumor metastasis evaluation. (**d**) T-SNE maps of the flow cytometric results on the expression of CD3, CD8, CD107a, GZMB, IFN-γ and TNF-α in spleen lymphocytes. (**e-h**) Quantifications of the percentages of CD4 + T cells, CD8 + T cells, CD8 + CD69 + T cells and CD8 + CD25 + T cells extracted from spleen of mice from different treatment groups. (**i-l**) Serum quantifications of the cytokines including (**i**) IFN-γ, (**j**) IL-6, (**k**) TNF-α and (**l**) IL-2. (**e-l**) *n* = 3, biologically replicates. Data are presented as they are and mean ± s.d

For anti-metastasis investigation, twenty-five Balb/c mice were randomly allocated to five groups: Control, OMVs, MLipoAA, OMVs/MLipoAA and IFN-γ/MLipoAA groups. Primary CT26 colon tumor cells (5 × 10^5^ cells/site) were subcutaneously administered into the right flank of Balb/c mice on Day 0, followed by intravenous injection of the tumor cells (5 × 10^4^ cells/site) to Balb/c mice to establish a lung metastasis model (Day 3). OMVs or IFN-γ were injected on Day 7 to the mice from OMVs, OMVs/MLipoAA and IFN-γ/MLipoAA groups, followed by MLipoAA injection 24 h later to the mice from OMVs/MLipoAA and IFN-γ/MLipoAA groups (Fig. 7a). On day 20, mice were sacrificed and the lung tissues were collected and fixed with Bouin’s solution for metastatic nodes identification (Fig. 7b). We observe a reduction of the counts of lung metastatic nodes for mice from OMVs or MLipoAA groups. Mice from the combinational OMVs/MLipoAA group exhibit complete inhibition of the lung metastases, as revealed by the full-view microscopic images of H&E-stained lung sections (Fig. 7c). Specifically, 35 nodes were counted in the control group, the node counting for the lung of mice from OMVs, MLipoAA, OMVs/MLipoAA and IFN-γ/MLipoAA groups are 10, 16, 1 and 11 respectively (Figure S31). To investigate the antitumor host immunity, T lymphocytes in spleens were examined and visualized using t-SNE analysis, presenting the overview of the T cells clusters annotated as CD3 + lymphocytes, CD3 + CD4 + IFN-γ lymphocytes, CD3 + CD4 + CD8 + TNF-α + IFN-γ + CD107a + lymphocytes, CD3 + CD8 + IFN-γ + Gzmb + lymphocytes. Enhanced T cell activation could be concluded for the spleen of mice from OMVs/MLipoAA group, as compared to control and other treatment groups (Fig. 7d, Figure S32). From the results, the populations of CD4+, CD8+, CD8 + CD69+, CD8 + CD25+, CD8 + GzmB+, and CD8 + CD107 + T cells from OMVs/MLipoAA group were upregulated, ranking the highest among the control group and other treatment groups (Fig. 7e-h, Figure S33). Besides, the upregulated secretion of IFN-γ, IL6, TNF-α and IL-2 cytokines could further facilitate the killing performance of CTLs, amplifying the in vivo adaptive antitumor immunity effectively to combat distant metastatic tumors (Fig. 7i-l).

## Conclusion

In summary, focusing on the immunosuppression condition induced by in vivo ferroptosis therapy by imidazole ketone erastin, the present work reports the pre-activation of the T cell immunity by engineered ClyA-encoded OMVs administration, optimally at dose of 1.25 µg and an elongated treatment period of 18 h. In combination with the constructed hybridized arachidonic acid containing nanovesicles (MLipoAA), pre-activation of the T cell immunity could effectively direct tumor cell ferroptosis mediated by IFN-γ-Acsl4 nexus, inducing prominent tumor destruction with immunopotentiation. The combinational ferroptotic therapy could also show inhibitory effects against tumor recurrence and metastasis, in surgical dissection-recurrence model and lung metastasis model respectively. The present work highlights the advances of pre-activation of T cell immunity for tumor ferroptosis direction, remodeling the immunosuppression microenvironment induced by traditional ferroptotic therapy.

## Supplementary Information

Supplementary Material 1.

## Data Availability

No datasets were generated or analysed during the current study.

## References

[1] Stockwell BR, Jiang X. The chemistry and biology of ferroptosis. Cell Chem Biol. 2020;27:365–75. 32294465 10.1016/j.chembiol.2020.03.013 PMC7204503

[2] Wang K, et al. Emerging roles of ferroptosis in cardiovascular diseases. Cell Death Discov. 2022;8:394. 10.1038/s41420-022-01183-2 36127318 10.1038/s41420-022-01183-2 PMC9488879

[3] Greenough MA, et al. Selective ferroptosis vulnerability due to Familial Alzheimer’s disease presenilin mutations. Cell Death Differ. 2022;29:2123–36. 35449212 10.1038/s41418-022-01003-1 PMC9613996

[4] Zhou Q, et al. Ferroptosis in cancer: from molecular mechanisms to therapeutic strategies. Signal Transduct Target Ther. 2024;9:55. 38453898 10.1038/s41392-024-01769-5 PMC10920854

[5] Hou DY, et al. In vivo assembly enhanced binding effect augments tumor specific ferroptosis therapy. Nat Commun. 2024;15:454. 38212623 10.1038/s41467-023-44665-2 PMC10784468

[6] Dixon SJ, et al. Ferroptosis: an iron-dependent form of nonapoptotic cell death. Cell. 2012;149:1060–72. 22632970 10.1016/j.cell.2012.03.042 PMC3367386

[7] Wiernicki B, et al. Cancer cells dying from ferroptosis impede dendritic cell-mediated anti-tumor immunity. Nat Commun. 2022;13:3676. 35760796 10.1038/s41467-022-31218-2 PMC9237053

[8] Chen R, Kang R, Tang D. The mechanism of HMGB1 secretion and release. Exp Mol Med. 2022;54(2):91–102. 35217834 10.1038/s12276-022-00736-w PMC8894452

[9] Zhang Y, et al. Imidazole ketone Erastin induces ferroptosis and slows tumor growth in a mouse lymphoma model. Cell Chem Biol. 2019;26:623–e633629. 30799221 10.1016/j.chembiol.2019.01.008 PMC6525071

[10] Dolma S, et al. Identification of genotype-selective antitumor agents using synthetic lethal chemical screening in engineered human tumor cells. Cancer Cell. 2003;3:285–96. 12676586 10.1016/s1535-6108(03)00050-3

[11] Dang Q, et al. Ferroptosis: a double-edged sword mediating immune tolerance of cancer. Cell Death Dis. 2022;13:925. 36335094 10.1038/s41419-022-05384-6 PMC9637147

[12] Binnewies M, et al. Understanding the tumor immune microenvironment (TIME) for effective therapy. Nat Med. 2018;24:541–50. 29686425 10.1038/s41591-018-0014-x PMC5998822

[13] Polak R, Zhang ET, Kuo CJ. Cancer organoids 2.0: modelling the complexity of the tumour immune microenvironment. Nat Rev Cancer. 2024;24:523–39. 38977835 10.1038/s41568-024-00706-6

[14] Zheng Y, et al. The crosstalk between ferroptosis and anti-tumor immunity in the tumor microenvironment: molecular mechanisms and therapeutic controversy. Cancer Commun. 2023;43:1071–96. 10.1002/cac2.12487 PMC10565387 37718480

[15] Kim R, et al. Ferroptosis of tumour neutrophils causes immune suppression in cancer. Nature. 2022;612:338–46. 36385526 10.1038/s41586-022-05443-0 PMC9875862

[16] Yang WS, et al. Regulation of ferroptotic cancer cell death by GPX4. Cell. 2014;156:317–31. 10.1016/j.cell.2013.12.010 24439385 10.1016/j.cell.2013.12.010 PMC4076414

[17] Wang D, Dubois RN. Eicosanoids and cancer. Nat Rev Cancer. 2010;10:181–93. 20168319 10.1038/nrc2809 PMC2898136

[18] Kalinski P. Regulation of immune responses by prostaglandin E2. J Immunol. 2012;188:21–8. 22187483 10.4049/jimmunol.1101029 PMC3249979

[19] Li H, et al. Regulation of T helper cell subsets by cyclooxygenases and their metabolites. Prostaglandins Other Lipid Mediat. 2013;104–105:74–83. 23201570 10.1016/j.prostaglandins.2012.11.002 PMC3620713

[20] Drijvers JM, et al. Pharmacologic screening identifies metabolic vulnerabilities of CD8(+) T cells. Cancer Immunol Res. 2021;9:184–99. 33277233 10.1158/2326-6066.CIR-20-0384 PMC7864883

[21] Kim R, et al. Ferroptosis of immune cells in the tumor microenvironment. Trends Pharmacol Sci. 2023;44:542–52. 37380530 10.1016/j.tips.2023.06.005

[22] Xue Y, et al. Intermittent dietary methionine deprivation facilitates tumoral ferroptosis and synergizes with checkpoint blockade. Nat Commun. 2023;14:4758. 37553341 10.1038/s41467-023-40518-0 PMC10409767

[23] Liu J, et al. Ultrathin clay Nanoparticles-Mediated mutual reinforcement of ferroptosis and cancer immunotherapy. Adv Mater. 2024;36:e2309562. 37939375 10.1002/adma.202309562

[24] Kim O, et al. Bacterial outer membrane vesicles suppress tumor by interferon-γ-mediated antitumor response. Nat Commun. 2017;8(1):616. 10.1038/s41467-017-00729-8 28931823 10.1038/s41467-017-00729-8 PMC5606984

