# Supplementary material for: Pre-activation of T cell immunity potentiates ferroptotic cell death through arachidonic acid hybridized nanovesicles
Source: J Nanobiotechnology. 2025 Nov 18;23:718. doi: 10.1186/s12951-025-03797-x (PMC12625620; doi:10.1186/s12951-025-03797-x)
Supplement: Supplementary file 1 — Supplementary Material 1. [file 12951_2025_3797_MOESM1_ESM.docx]

Supporting Information for

Pre-Activation of T cell Immunity Potentiates Ferroptotic Cell Death Through Arachidonic Acid Hybridized Nanovesicles

Qi Lyu,^1#^ Chang Liu,^2#^ Shaoyue Li,^2#^ Dandan Shan,^2^ Hong Han, ^1*^ Liying Wang,^2*^ Huixiong Xu^1*^

^1^ Department of Ultrasound, Zhongshan Hospital, Institute of Ultrasound in Medicine and Engineering, Fudan University, Shanghai 200032, P. R. China

xu.huixiong@zs-hospital.sh.cn (Prof. H. Xu)

^2^ Department of Medical Ultrasound, Shanghai Tenth People’s Hospital; Shanghai Frontiers Science Center of Nanocatalytic Medicine, School of Medicine, Tongji University, Shanghai, 200072, P. R. China

Han.hong@zs-hospital.sh.cn (Prof. H. Han); ly_wang@tongji.edu.cn (Prof. L. Wang)

1. Supplementary Methods

**Materials.** Arachidonic acid was purchased from Sigma-Aldrich. Dimethyl sulfoxide (DMSO) and trichloromethane (CHCl_3_) were purchased from the Sinopharm Chemical Reagent Co. Dipalmitoyl phosphatidylcholine (DPPC), 1,2-distearoyl-sn-glycero-3- phosphoethanolamine-N-[amino(polyethylene glycol)2000] (DSPE-PEG-2000), and cholesterol was purchased from Xi’an Ruixi Biological Technology Co., Ltd. Bicinchoninic acid assay kit (BCA kit), DAPI, and Mitochondrial membrane potential assay kits (with JC-1) were purchased from Beyotime Biotechnology Co., Ltd. C11-BODIPY probe was purchased from Thermo Invitrogen. The spleen lymphocyte Isolation Kit was purchased from Solarbio Science & Technology Co., Ltd. The CCK-8 assay kit was purchased from Dojindo Laboratories, Japan. Antibodies and inhibitors are listed on the **Table S1**.

Table S1. Information of reagents used in this work.

| Reagent | Source | Identifier |
| --- | --- | --- |
| Antibodies | | |
| Anti-ACSL4 Antibody | Santa Cruz | Cat# sc-271800 |
| Anti-GPX4 Antibody | Cell Signaling Technology | Cat #52455 |
| APC-Cy7 Rat Anti-Mouse CD45(30-F11) | BD Pharmingen | Cat #557659 |
| BB700 Armenian Hamster Anti-Mouse CD3e(145-2C11) | BD Pharmingen | Cat #566494 |
| FITC Rat Anti-Mouse CD3 Molecular Complex(17A2) | BD Pharmingen | Cat #561798 |
| BV605 Rat Anti-Mouse CD4 (RM4-5) | BD Pharmingen | Cat #563151 |
| BV786 Rat Anti-Mouse CD8a (53-6.7) | BD Pharmingen | Cat #563332 |
| FITC Rat Anti-CD11b(M1/70) | BD Pharmingen | Cat #557396 |
| BV421 Rat Anti-Mouse F4/80 (T45-2342) | BD Pharmingen | Cat #565411 |
| APC Rat anti-Mouse CD86 (GL1) | BD Pharmingen | Cat #558703 |
| PE Rat Anti-Mouse CD206 (Y17-505) | BD Pharmingen | Cat #568273 |
| Fixable Viability Stain 510 | BD Pharmingen | Cat #564406 |
| PE Rat Anti-Mouse CD25 (PC61) | BD Pharmingen | Cat # 561065 |
| APC Hamster Anti-Mouse CD69 (H1.2F3) | BD Pharmingen | Cat # 560689 |
| APC Rat Anti-Mouse IFN-γ (XMG1.2) | BD Pharmingen | Cat # 562018 |
| Granzyme B Monoclonal Antibody (NGZB), PE, eBioscience™ | Thermo Fisher | Cat # 12-8898-80 |
| Brilliant Violet 421™ anti-mouse CD107a (LAMP-1) Antibody | Biolegend | Cat # 121618 |
| Chemicals, peptides, and recombinant proteins | | |
| Arachidonic acid | Sigma-Aldrich | Cat # A3611 |
| Ferrostatin-1 | MedChemExpress | Cat # HY-100579 |
| Necrostatin-1 | MedChemExpress | Cat # HY-15760 |
| Z-VAD-FMK | MedChemExpress | Cat # HY-16658B |
| 3-Methyladenine | MedChemExpress | Cat # HY-19312 |
| Recombinant Mouse IFN gamma | novoprotein | Cat # C746 |
| BODIPY 581/591 C11 (Lipid Peroxidation Sensor) | Thermo Fisher | Cat# D3861 |
| Mouse IFN-γ High Sensitivity ELISA Kit | MULTI SCIENCES | Cat# EK280HS |

**Characterizations.** Ultrasonic cell disruption was carried out on DH92‐IIN, LAWSON, China. Bio-TEM images were obtained on a HITACHI H-7800. Confocal microscopic images were obtained on an Olympus BX51. Flow cytometry analysis was carried out on CytoFLEX LX. Ultrasonic elastography of subcutaneous tumor xenografts was performed on Sonosite P25 Elite.

**Preparation of arachidonic acid hybridized nanovesicles.** Arachidonic acid hybridized liposome (designated as LipoAA) was synthesized via a typical reverse evaporation method. Arachidonic acid (0.922 g) was firstly dissolved in DMSO (1 mL) and then added into CHCl_3_ solution containing 12 mg 1,2-Dihexadecanoyl-rac-Glycero-3-Phosphocholine (DPPC), 4 mg DSPE-PEG-2000 and 4 mg cholesterol with a fixed weight ratio of 3:1:1. The following evaporation on a rotary evaporator at 100 mbar and 100 rpm at 60 ^o^C for 1 h was carried out. After that, the pressure was decreased to 0 mbar, and the mixture was rotated and evaporated for 6 h, aiming to completely remove the solvent and obtain the lipid thin film. The lipid film was redissolved in 5 mL PBS (pH = 7.4). Following that, the mixture was treated by the ultrasonic cell disruptor (power: 60 W; duty cycle: 50 %) for 1 minute. Afterward, the mixture was centrifuged at 12000 rpm for 30 min at 4 °C. Ultimately, the precipitate was redispersed into 10 mL PBS (pH 7.4) for further use.

The CT26 tumor cell membrane was collected using Mem-PER^TM^ Plus Membrane Protein Extraction Kit (Thermo Scientific) and subsequently hybridized onto LipoAA using a liposome extruder (LP-15, Avestin) at a 1:1 mass ratio, following the manufacturer's protocols.

**Strain construction and isolation of outer membrane vesicle.** Plasmid containing the T7 promotor, ClyA-sfGFP, Kanamycin Resistance Protein, and Terminator was constructed. The competent Rosetta (DE3) strain was transformed with the constructed plasmid through heat shock. The obtained engineered stain was culture in LB medium at 37 ^o^C for 24 h with shaking at 120 rpm. Supernatants were collected after centrifugation at 7000 rpm for 10 min to remove the bacterial cells, followed by centrifugation using 100 kDa ultrafiltration tube. The collected OMVs were further washed with PBS and stored at -20 ^o^C. The total protein content was evaluated by the BCA kit. Protein sequence for ClyA is as follow: MTEIVADKTVEVVKNAIETADGALDLYNKYLDQVIPWQTFDETIKELSRFKQEYSQAASVLVGDIKTLLMDSQDKYFEATQTVYEWCGVATQLLAAYILLFDEYNEKKASAQKDILIKVLDDGITKLNEAQKSLLVSSQSFNNASGKLLALDSQLTNDFSEKSSYFQSQVDKIRKEAYAGAAAGVVAGPFGLIISYSIAAGVVEGKLIPELKNKLKSVQNFFTTLSNTVKQANKDIDAAKLKLTTEIAAIGEIKTETETTRFYVDYDDLMLSLLKEAAKKMINTCNEYQKRHGKKTLFEVPEV.

**Cell Culture.** Murine colon cancer cells (CT26 cells) were purchased from the National Collection of Authentical Cell Cultures. The cells were cultured in RPMI 1640 medium supplemented with 10 % fetal bovine serum (FBS) and 1 % Penicillin–Streptomycin in 5 % CO_2_ humidified atmosphere at 37 ^o^C. T cells isolated from spleens were incubated under the same conditions. Bone marrow-derived BMDCs were also incubated with 1640 medium containing additional GM-CSF (1000 U/mL) and IL-4 (1000 U/mL) at 37 ^o^C in a humidified atmosphere with 5 % CO_2_.

**T cell isolation and culture.** To prepare single-cell level T cells suspensions, the spleen tissue was dissected from six-weeks-old Balb/c mice, placed in a sterile plastic dish, then minced and grounded on 70 μm cell strainers. The lymphocytes were then isolated from spleen tissue using spleen lymphocyte Isolation Kit. Isolated lymphocyte cells were washed with PBS, and resuspended in RPMI 1640 culture medium at 1×10^7^ cells/mL.

**Evaluation of T cell activation.** Isolated murine lymphocytes were washed, and resuspended at 1×10^6^ cells/well in 6-well plates with RPMI 1640 medium containing OMVs at varied doses (0, 1.25, 2.5 μg/well) at 37 ^o^C for 6 h or 18 h respectively. Cells were then harvested and centrifuged at 1000 rpm for 5 min. After washing with PBS, cells were stained with anti-FITC-CD3, and anti-APC-CD69 at 4 ^o^C for 30 min. T cells were washed and resuspended in 1 mL of freshly prepared Fix/Perm solution at 4 ^o^C overnight. After rinsing with 1 mL Perm/Wash buffer twice, cells were stained with anti-APC-IFN-γ, anti-PE-GzmB, and anti-BV421-CD107 antibodies. Subsequently, cells were washed, and fixed in 4 % formaldehyde, followed by flow cytometry detection. Besides, to quantify the IFN-γ produced by activated T cells, supernatants after OMVs co-incubations were collected for ELISA.

**Cellular toxicity investigations.** Cellular cytotoxicity profiles of different material components are evaluated using CCK-8 assay. Typically, CT26 cells were seeded into a 48-well plate (8000 cells/well). After overnight incubation, fresh medium containing MLipoAA at varied concentrations (120, 100, 80, 60, 40, and 0 μM) with or without IFN-γ (10 ng/mL) was added and incubated for 24 h. To verify the type of programmed cell death pathway, Fer-1 (working concentration: 2 μM), Z-VAD (working concentration: 40 μM), Nec-1 (working concentration: 30 μM), and 3-MA (working concentration: 5 mM) were also added to co-incubate with the cells. Afterward, a CCK-8 working solution (10 vol%) was added into the medium for 4 h. Optical absorbance was measured at 450 nm using microplate reader.

For OMVs therapeutics, T cells were pre-activated with OMVs at varied concentrations (0, 1.25, 2.5 μg/well) for varied time points (6, 12 and 18 h). The supernatants were then added into CT26 cell culture and incubated for 24 h. Then, CT26 cells were washed twice with PBS, and CCK-8 reagent was added to each well to evaluate the cell viability.

For combinational OMVs/MLipoAA therapeutics, CT26 cells in 24 well plates were further incubated with supernatants of T cells after OMV stimulation at varied concentrations (0, 1.25, 2.5 μg per 10^6^ T cells per well) and time points (6, 12, and 18 h), with or without MLipoAA (60 μM). Fer-1 (working concentration: 2 μM), Z-VAD (working concentration: 40 μM), Nec-1 (working concentration: 30 μM), and 3-MA (working concentration: 5 mM) were additionally added to the cells. At the end of 24 h incubation, a CCK-8 working solution was added to test cellular toxicity.

**C11-BODIPY staining.** CT26 tumor cells (10^6^ cells/well) were seeded in a 6-well plate. After being treated with IFN-γ (10 ng/ml), MLipoAA (60 μM), MLipoAA (60 μM) / IFN-γ (10 ng/mL), MLipoAA (60 μM)/T(sup) (1.25 μg OMVs per 10^5^ T cells), MLipoAA (60 μM) / T(sup) (1.25 μg OMVs per 10^5^ T cells)/Fer-1 (2 μM) for 48 h, cells were harvested after trypsinization for 2 min at 37 ^o^C. Then the cells were washed with cold PBS and resuspended in 1 mL PBS, containing 5 mM C11-BODIPY probe (E_x_/E_m_: 581/591), followed by incubation a 37 ^o^C for 15 min. Subsequently, the cells were washed and resuspended in 200 μL cold PBS for cellular fluorescence imaging and flow cytometry analysis.

**Western blot.** CT26 cells were seeded in a 6-well plate (1 × 10^6^ cells per well) and treated with RPMI 1640 medium containing IFN-γ (10 ng/ml), MLipoAA (60 μM), MLipoAA (60 μM) / IFN-γ (10 ng/mL), MLipoAA (60 μM) / T(sup) (1.25 μg OMVs per 10^5^ T cells), MLipoAA (60 μM) / T(sup) (1.25 μg OMVs per 10^5^ T cells)/Fer-1 (2 μM) respectively for 24 h. After trypsin-EDTA digestion and centrifugation, cells were collected and washed twice with cold PBS. Total protein was collected after RIPA treatment, quantified using a BCA assay kit, and boiled with loading buffer for western blot assays.

**Animal experiment.** Six-week-old Balb/c mice were purchased from Shanghai Model Organisms Center, Inc (Shanghai, China). Pathogen-free conditions were maintained for the mice. All animal experiments were in accordance with the guidelines of the Reginal Ethics Committee for Animal Experiments and were approved by the administrative committee of laboratory animals of Shanghai Tenth Peoples Hospital (approval number: SHDSYY-2023-6600).

**In vivo anti-tumor effect against subcutaneous CT26 tumor xenografts.** Female Balb/c mice at 6 weeks old were randomly divided into five groups (saline, OMVs, MLipoAA, OMVs / MLipoAA, IFN-γ/ MLipoAA) (OMVs: 5 μg per mice; MLipoAA: 2 μg per mice, IFN-γ: 2 μg per mice). These mice were subcutaneously injected with 10^6^ CT26 tumor cells on the right flank. When the volume of the tumor xenograft grew to 100 mm^3^, mice from OMVs, OMVs / MLipoAA and IFN-γ/ MLipoAA groups were injected with OMVs (i.p.) or IFN-γ (i.t.) (n = 5 for each group), followed by intravenous injection of MLipoAA at 24 h post. The tumor volume of the xenografts was recorded every two days using the formula: length × width^2^/2. When the tumor volume reached 1000 mm^3^, mice were euthanized according to the principles of animal protection and welfare. All mice were euthanized on day 15 and tumor tissues were collected and fixed in 4 % paraformaldehyde for H&E staining and immunofluorescence (IF) staining for CD45, CD8, and IFN-γ. Furthermore, western blot assays were performed using 50 mg tumor tissue with rabbit anti-ACSL (1/10000 dilution) and anti-GPX4 (1/10000 dilution) antibodies. WB assays were conducted according to standard protocols.

To evaluate the tumor-draining lymphocytes, tumor tissues were harvested and digested into a single cell suspension and stained with anti-FITC-CD3, anti-BV605-CD4, anti-BV786-CD8, anti-APC-CD86, and anti-PE-CD206. After being washed twice with cold PBS, cells were quantified using flow cytometry.

**In vivo anti-tumor effect against tumor recurrence model.** Six-week-old Balb/c mice were subcutaneously injected with 5 × 10^5^ CT26 tumor cells on the right flank of mice and were randomly divided into five groups (saline, OMVs, MLipoAA, OMVs/MLipoAA, IFN-γ/MLipoAA). When the volume of the tumor xenograft grew to 100 mm^3^, mice from OMVs, OMVs / MLipoAA and IFN-γ/ MLipoAA groups were injected with OMVs (i.p.) or IFN-γ (i.t.) (n = 5 for each group), followed by intravenous injection of MLipoAA at 24 h post. At 5 days post treatments, mice were performed with tumor removal surgery (remaining tumor volume of approximately 50 mm^3^). The tumor volume of the xenografts was recorded every two days using the formula: length × width^2^/2. To evaluate the tumor-draining lymphocytes, tumor tissues were harvested and digested into single cell suspensions, followed by staining with anti-FITC-CD3, anti-BV605-CD4, anti-BV786-CD8, anti-PE-CD25 and anti-APC-CD69 at 4°C for 30 min. Anti-APC-IFN-γ, anti-PE-GzmB and anti-BV421-CD107, anti-BV650-TNF-α were stained after cell permeabilization. After washed twice with PBS, cells were quantified using flow cytometry.

**In vivo anti-tumor effect against tumor pulmonary metastasis model.** The pulmonary metastasis model of colon carcinoma was established on the subcutaneous CT26 tumor-bearing Balb/c mice. The tumor-bearing mice were randomly divided into five groups (saline, OMVs, MLipoAA, OMVs/MLipoAA, IFN-γ/MLipoAA). They were injected with 10^6^ CT26 tumor cells *via* the tail vein. 7 days later, mice from OMVs, OMVs / MLipoAA and IFN-γ/ MLipoAA groups were injected with OMVs (i.p.) or IFN-γ (i.t.) (n = 5 for each group), followed by intravenous injection of MLipoAA at 24 h post. At Day 20, all mice were euthanized and the lungs were fixed into 4% paraformaldehyde for H&E staining. The spleens were collected for systemic immune analysis. Single-cell suspension of spleens was stained with membrane antibodies including anti-FITC-CD3, anti-BV605-CD4, anti-BV786-CD8, anti-PE-CD25, anti-APC-CD69 and intracellular antibodies including anti-APC-IFN-γ, anti-PE-GzmB and anti-BV421-CD107, anti-BV650-TNF-α as previously described. After washed twice with PBS, cells were quantified using flow cytometry.

Supplementary Figures

**
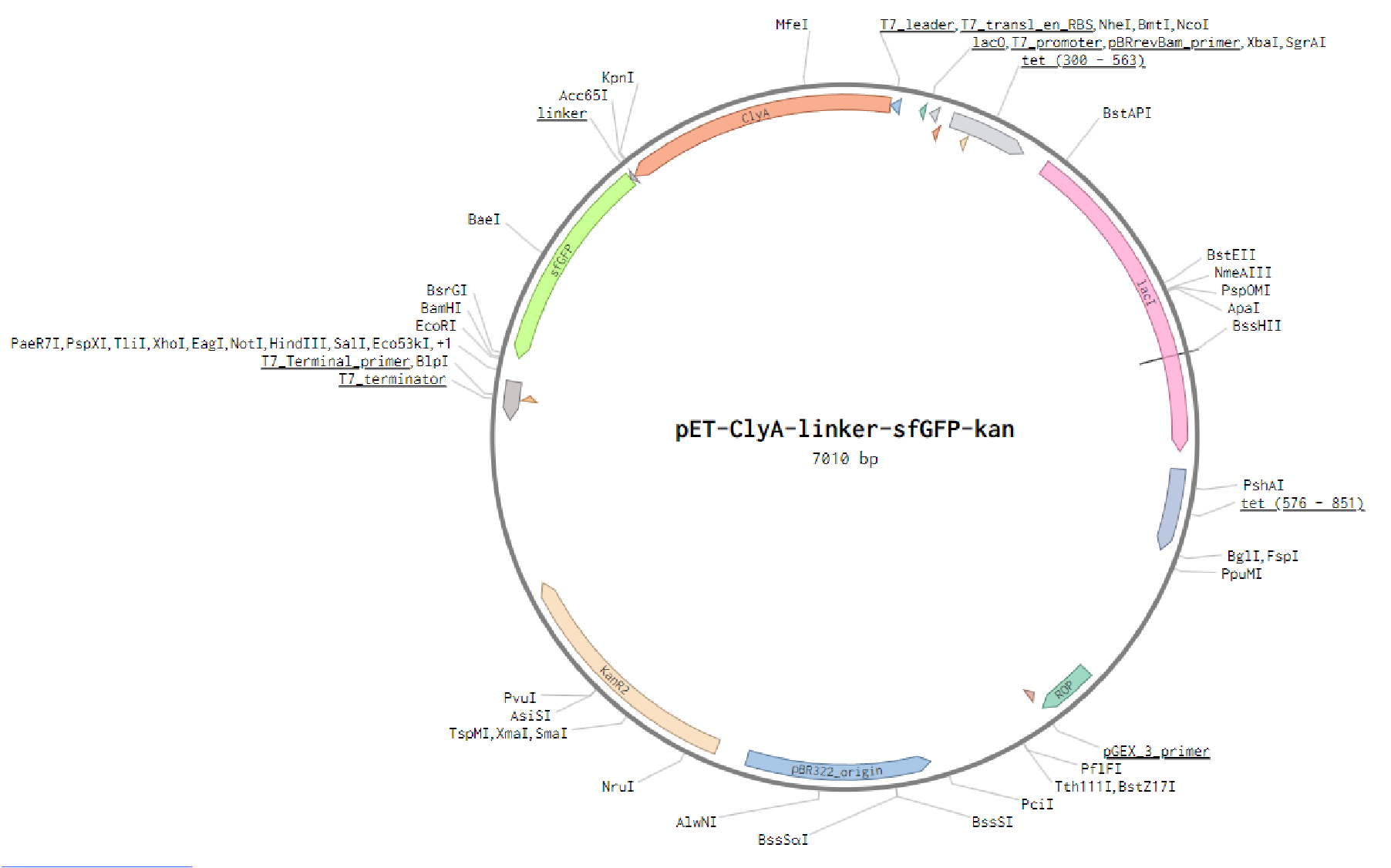
Figure S1.** The Complete DNA sequence of plasmid transformed inside Rosetta (DE3).


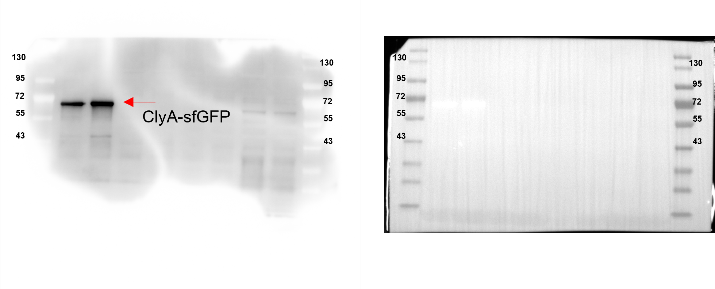


**Figure S2.** Western blot analysis of ClyA-sfGFP protein expression in the engineered Rosetta (DE3).


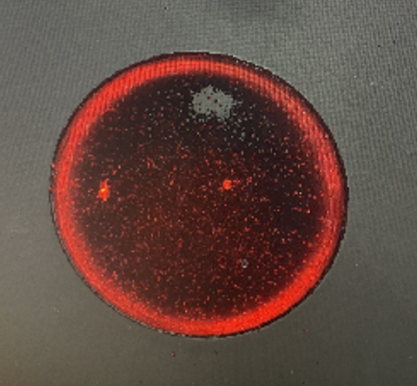


**Figure S3.** In vivo fluorescence imaging of the fluorescent bacterial colonies expressed on Kanamycin-containing LB agar plate.


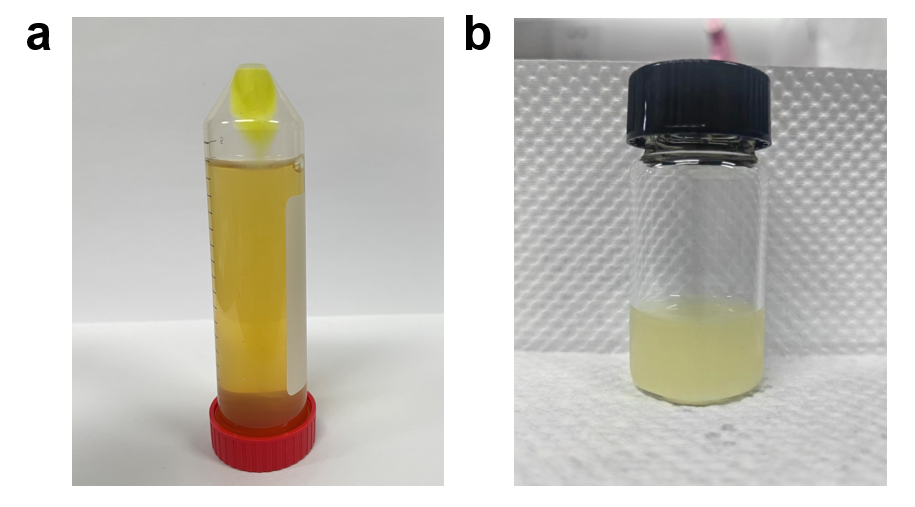


**Figure S4.** Digital photographs of (a) the pellet of engineered Rosetta (DE3) strain and (b) the derived OMVs suspension after ultrafiltration.


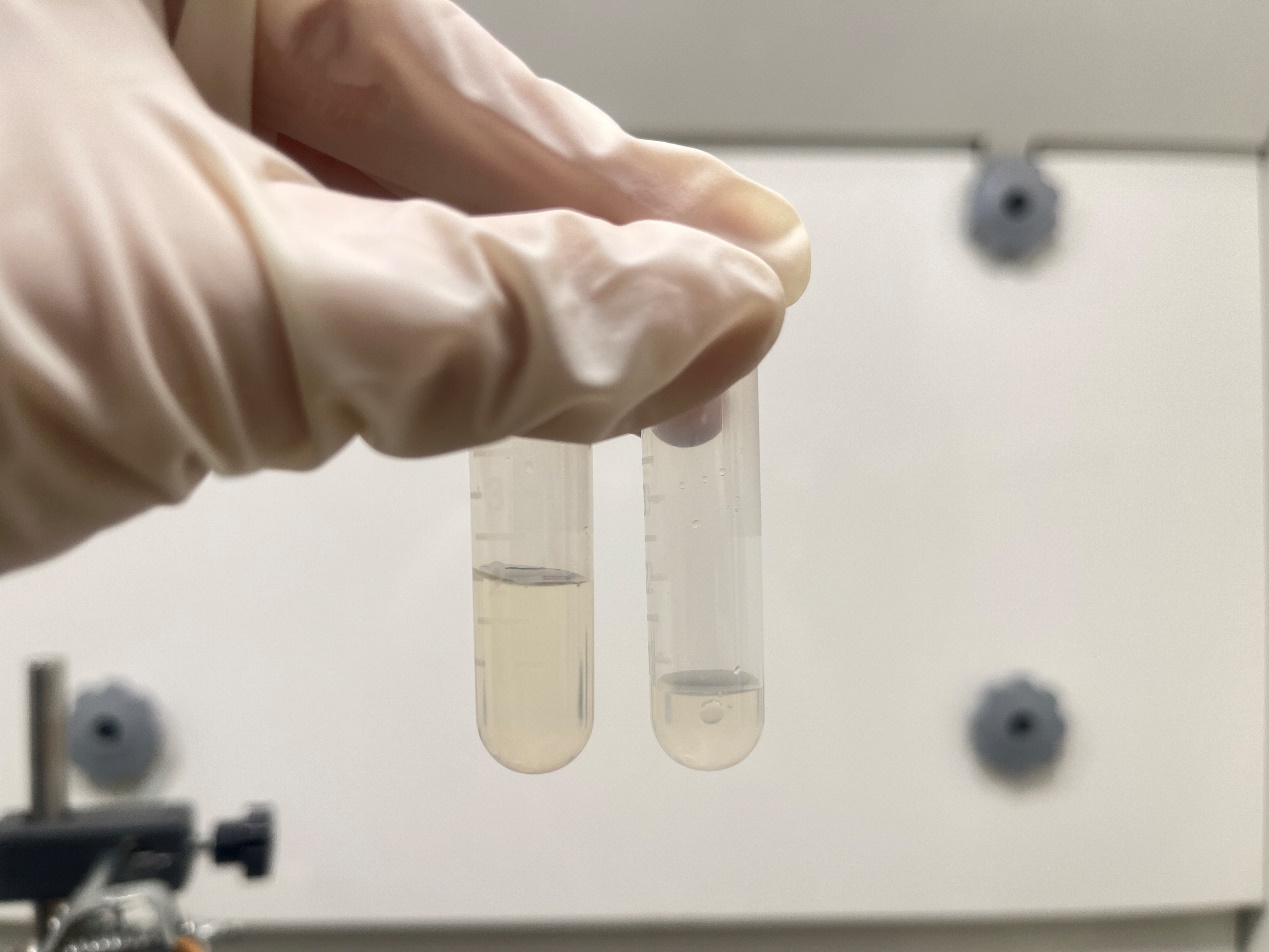


**Figure S5.** Digital photographs of OMVs (left) and WT-OMVs (right) quantified as 0.5mg/ml from 100ml LB medium with 1.5🞨10^10^ cells.


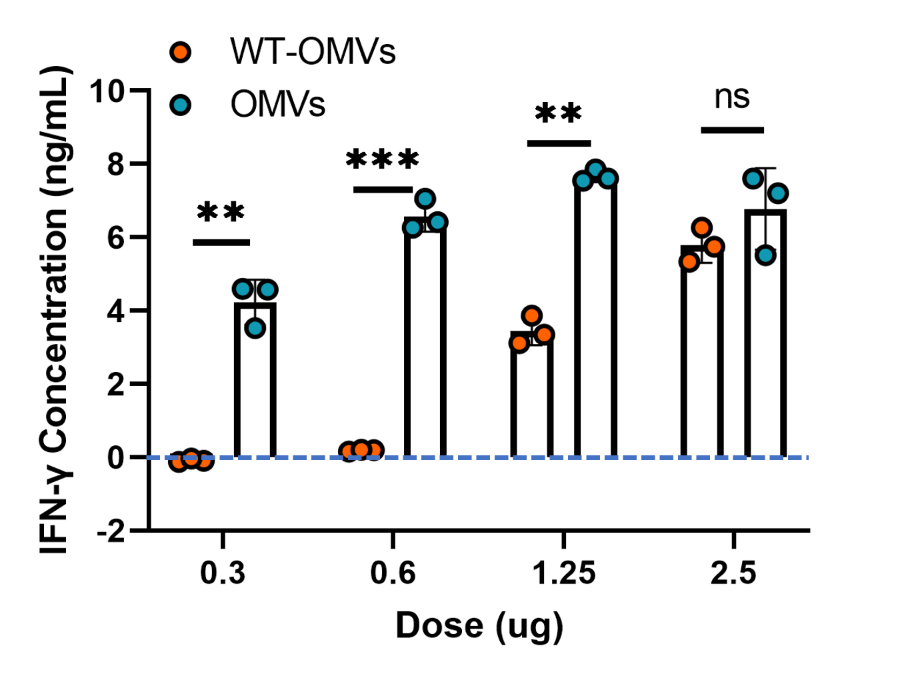


**Figure S6.** Quantitative IFN-γ expression of T cells after co-incubation with OMVs and WT-OMVs for 18 h at varied dose.


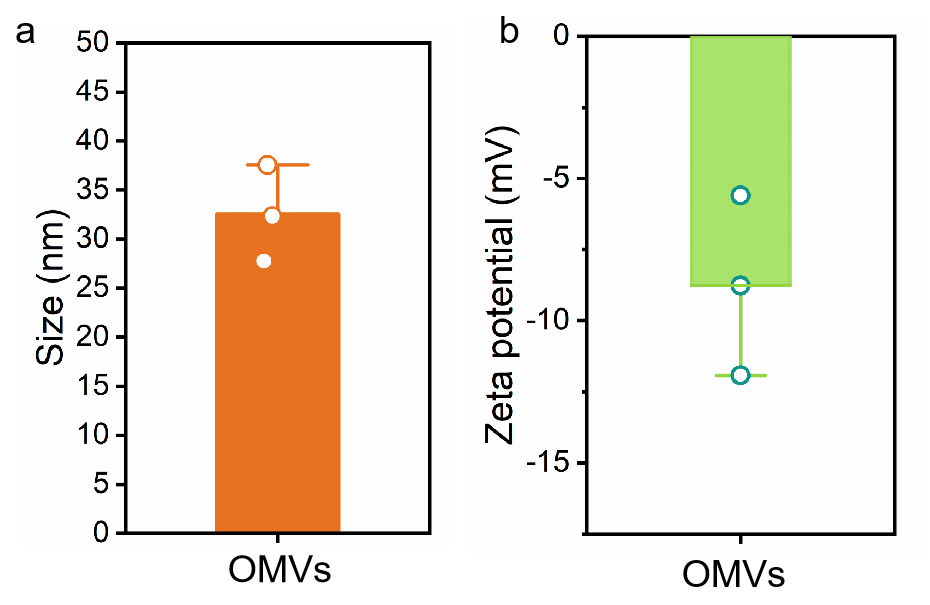


**Figure S7. a-b,** Hydrodynamic diameter (a) and zeta potential (b) of the purified OMVs.


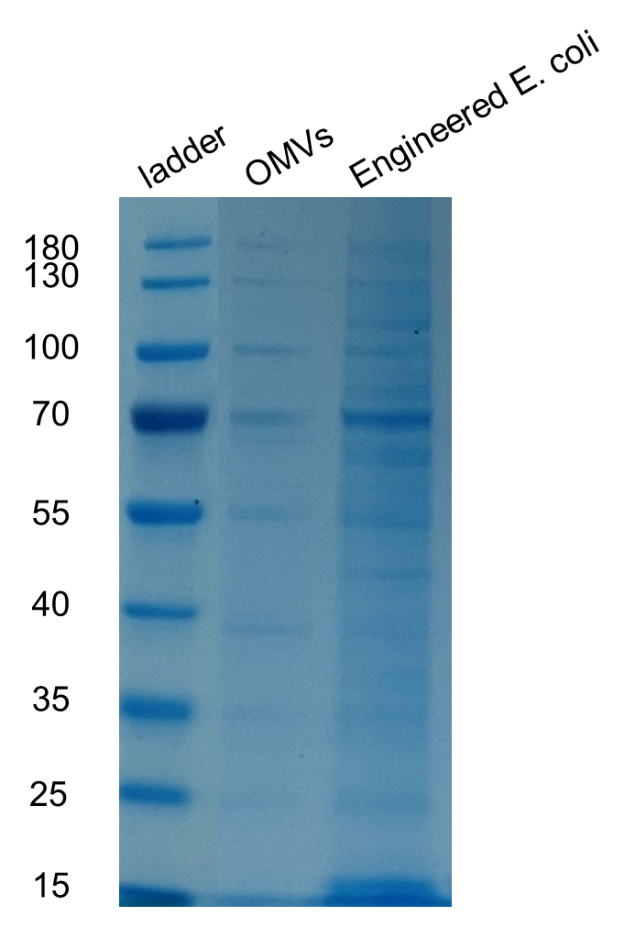


**Figure S8.** Coomassie brilliant blue staining of ClyA-OMVs and the engineered E. coli.


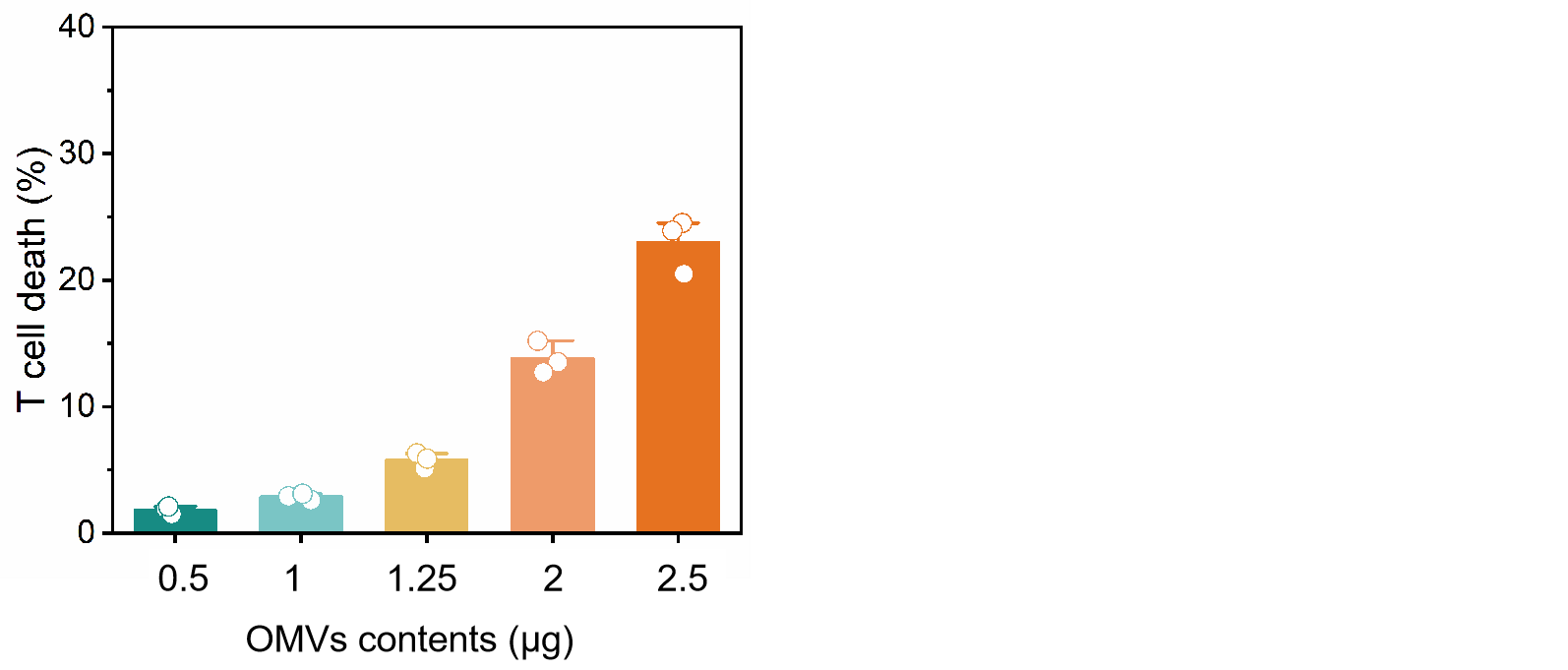


**Figure S9.** Cytotoxicity of T cells induced by OMVs at varied concentrations (0.5, 1, 1.25, 2, and 2.5 μg).


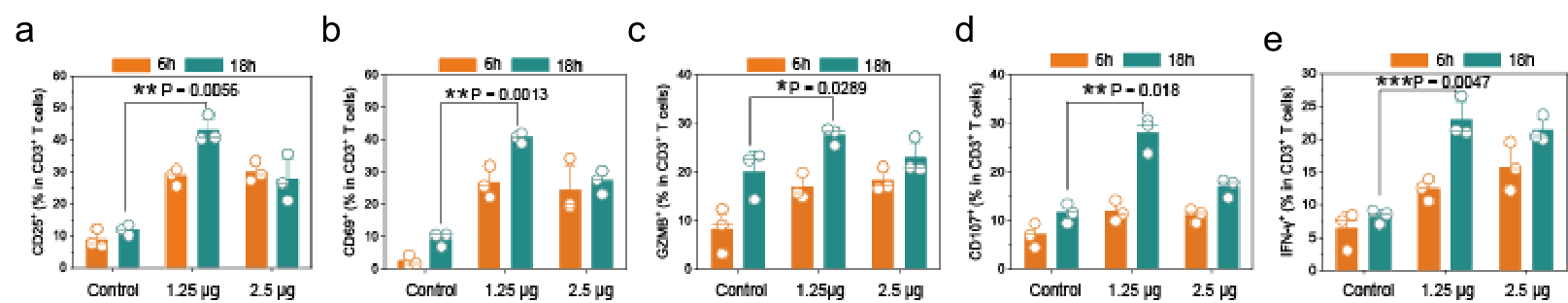


**Figure S10.** Statistical analysis of the flow cytometric results for Figure 2h-l.


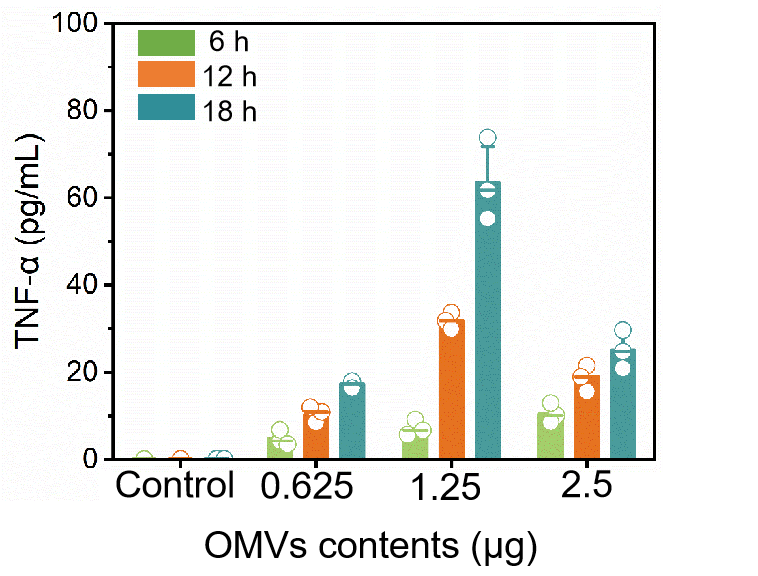


**Figure S11.** Quantitative TNF-α expression of T cells after OMVs stimulation at varied concentrations (0, 0.625, 1.25, and 2.5 μg) for 6 h, 12 h, and 18 h *via* ELISA.


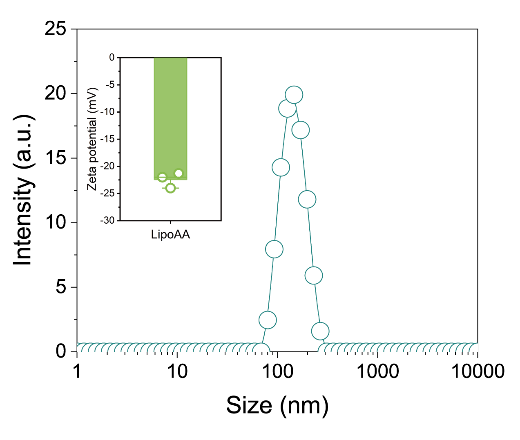


**Figure S12.** DLS spectrum and zeta potential of LipoAA.


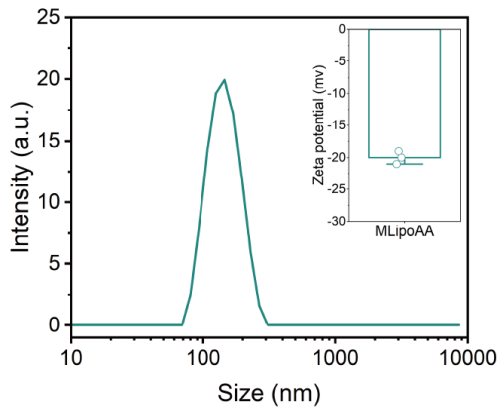


**Figure S13.** DLS spectrum and zeta potential of MLipoAA.


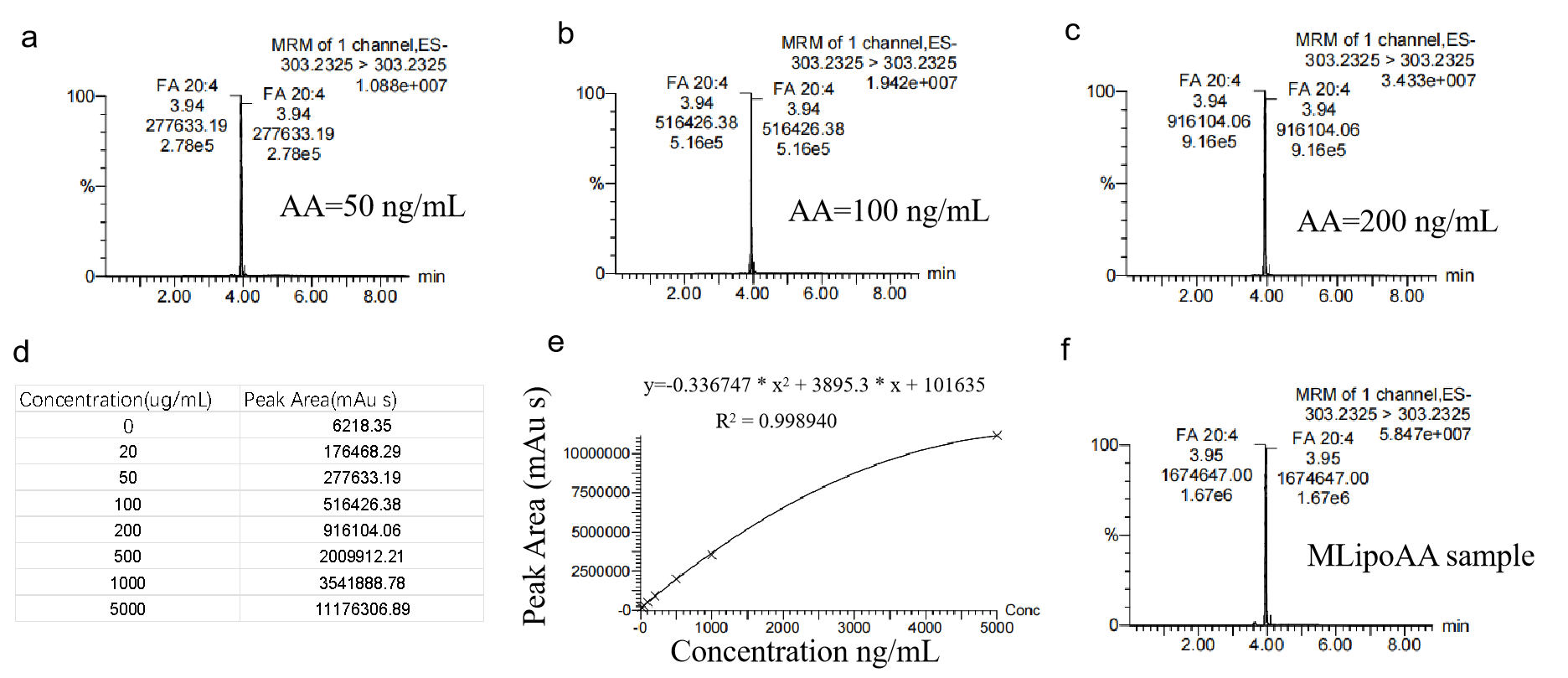


**Figure S14.** a-c, HPLC chromatogram of arachidonic acid at (a) 50 ng/mL, (b) 100 ng/mL, (c) 200 ng/mL. (d) Peak area profiles of the arachidonic acid standards (e) and their standard curve with linear fitting. (f) HPLC chromatogram of MLipoAA NPs.


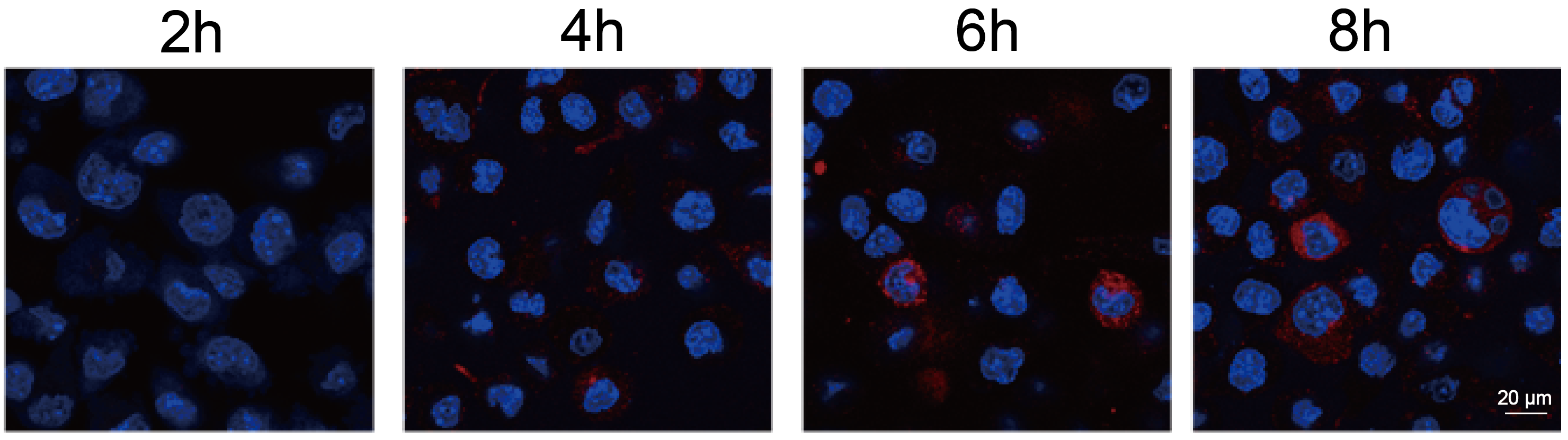


**Figure S15.** CLSM imaging of intracellular accumulation of DiR-stained MLipoAA.


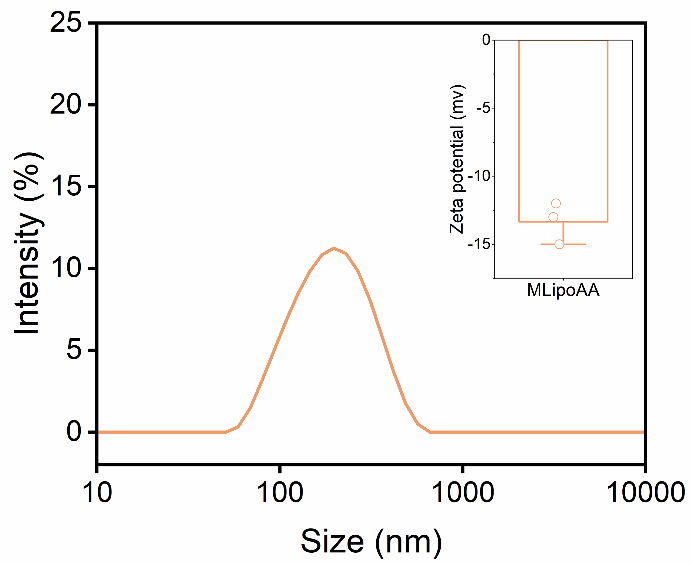


**Figure S16.** DLS spectrum and zeta potential of MLipoAA dispersed in PBS with pH of 5.0 for 12 h.


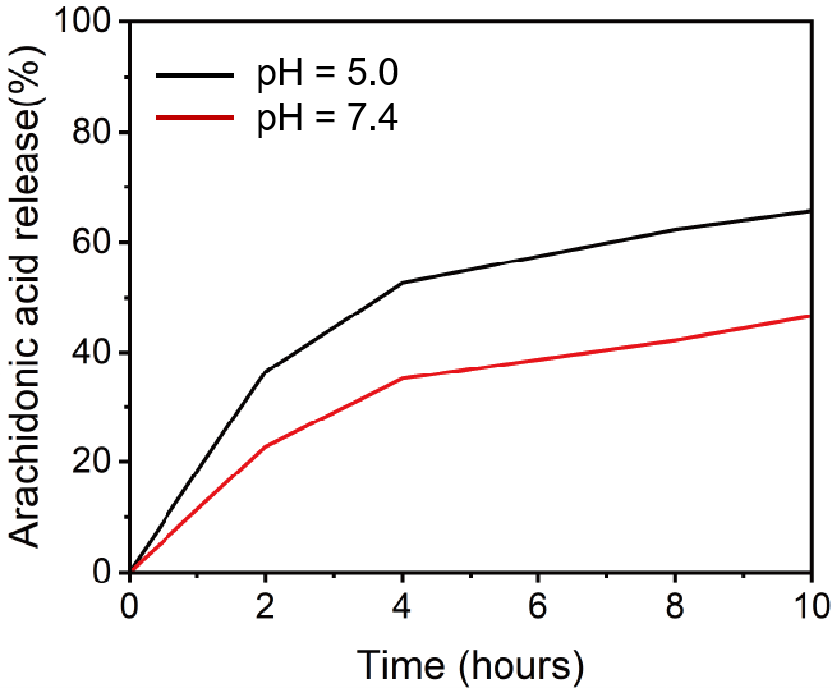


**Figure S17.** Release curve of arachidonic acid from MLipoAA at varied time points after dispersed in PBS with pH of 7.4 and 5.0.


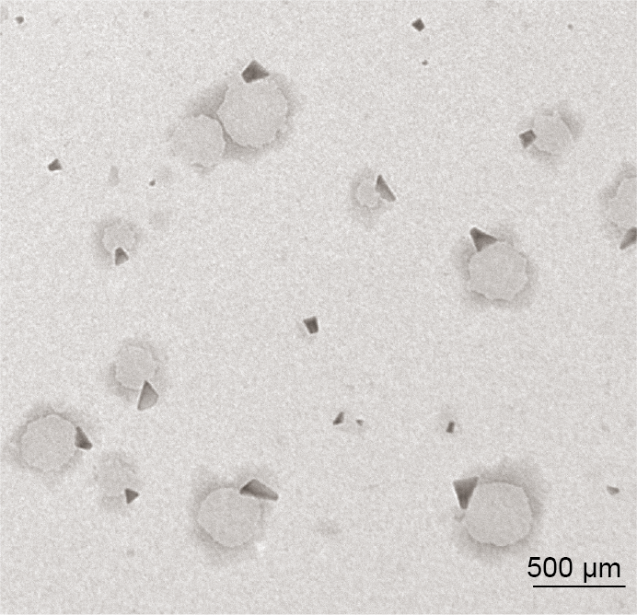


**Figure S18.** TEM image of MLipoAA after treatment in PBS with pH of 5.0 for 12 h.


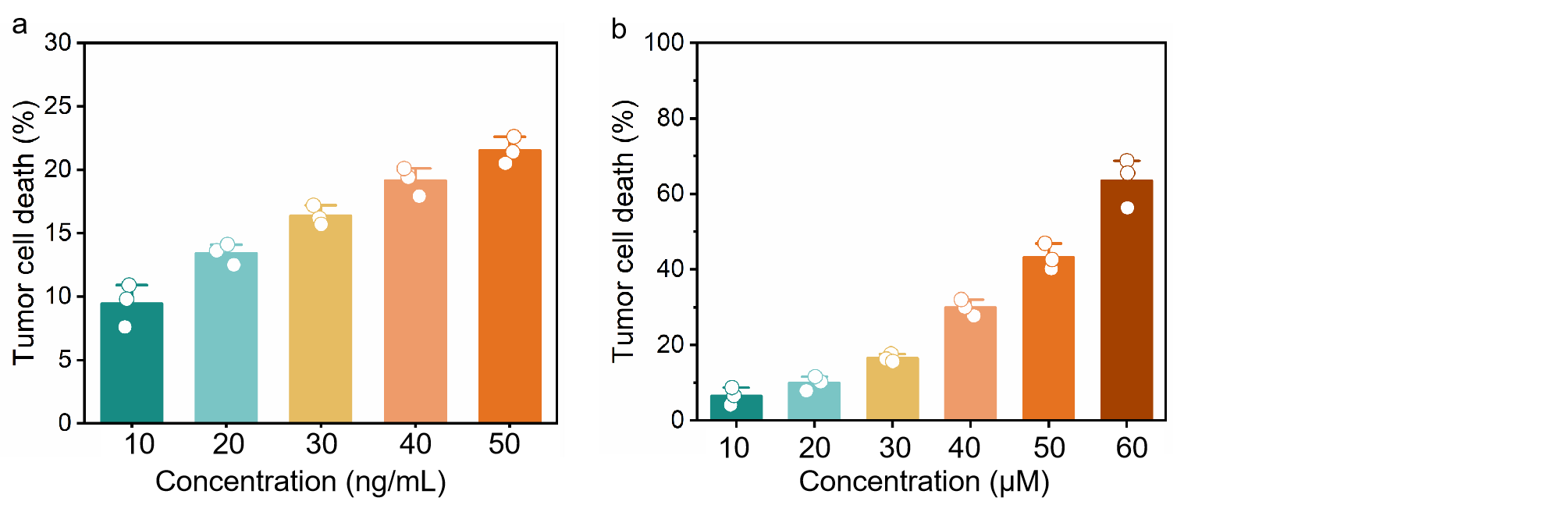


**Figure S19.** (a) Cytotoxicity of IFN-γ towards CT26 tumor cells at varied concentrations (10, 20, 30, 40, and 50 ng/mL); (b) Cytotoxicity of AA towards CT26 tumor cells at varied concentrations (10, 20, 30, 40, 50 and 60 μM).


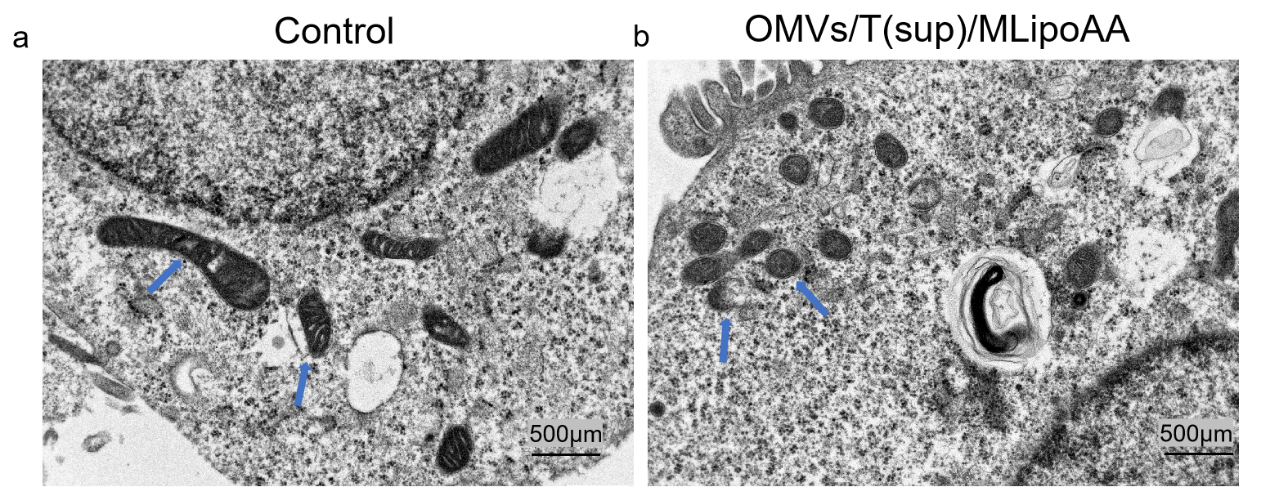


**Figure S20.** Bio-TEM images of CT26 cells after co-incubation with PBS and OMVs/T(sup)/MLipoAA, scale bar 500 μm.


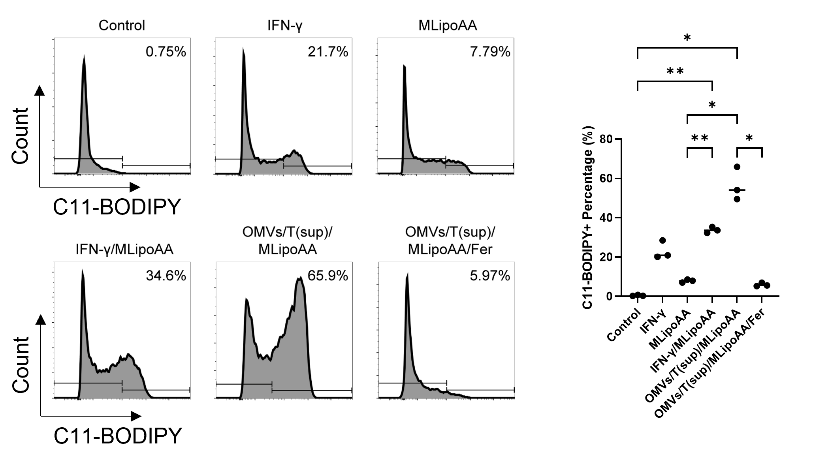


**Figure S21.** Flow cytometry results of C11-BODIPY-stained CT26 cells after co-incubation with PBS, IFN-γ, MLipoAA, IFN-γ/MLipoAA, OMVs/T(sup) /MLipoAA, and OMVs/T(sup) /MLipoAA /Fer.


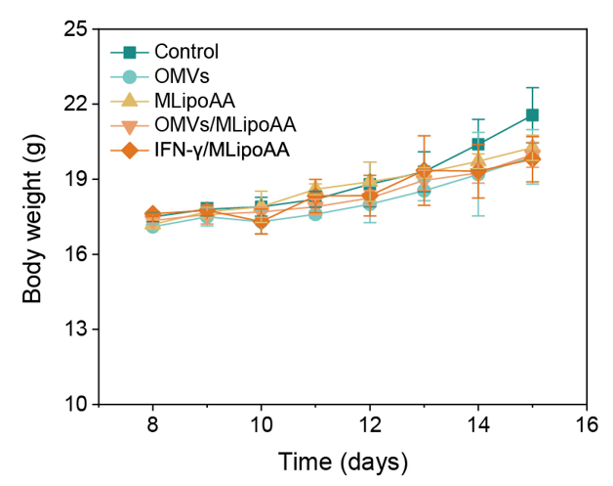


**Figure S22.** Body weight of mice in each group during the therapeutic timeframe, including control, OMVs, MLipoAA, OMVs/MLipoAA, IFN-γ/MLipoAA.


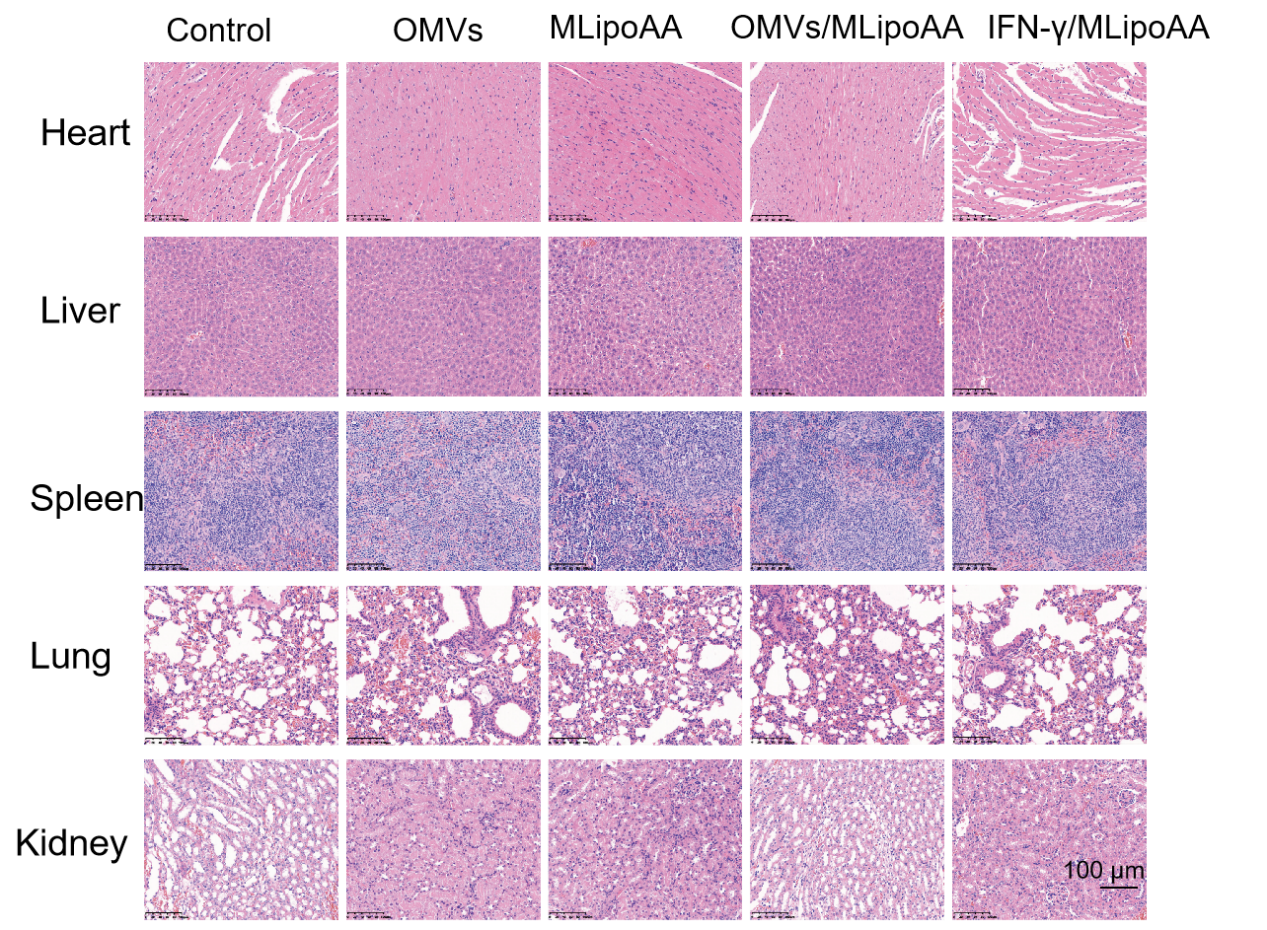


**Figure S23.** Microscopic images of H&E-stained major organs (heart, liver, spleen, lung, and kidney) of mice from different groups: Saline (Control), OMVs, MLipoAA, OMVs/MLipoAA, and IFN-γ/MLipoAA.


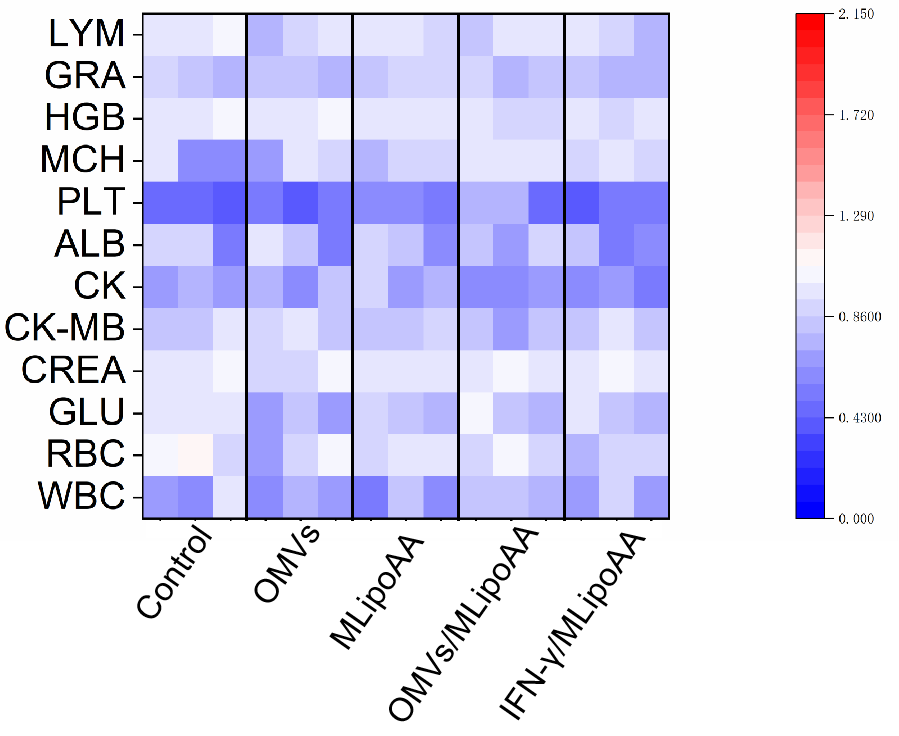


**Figure S24.** Plasma routine indices and blood chemical indices of mice from different treatment groups. Indices include white blood cells (WBC, 10^9^/L), lymphocyte (LYM, %), granulocytes (GRA, %), mean corpuscular hemoglobin (MCH, pg), red blood cells (RBC, 10^12^/L), platelet (PLT, 10^9^/L), albumin (ALB, g/L), creatine kinase (CK, U/L), creatine kinase-MB (CK-MB, U/L), blood creatinine (CREA, μmol/L) and glucose (GLU, mmol/L) markers.


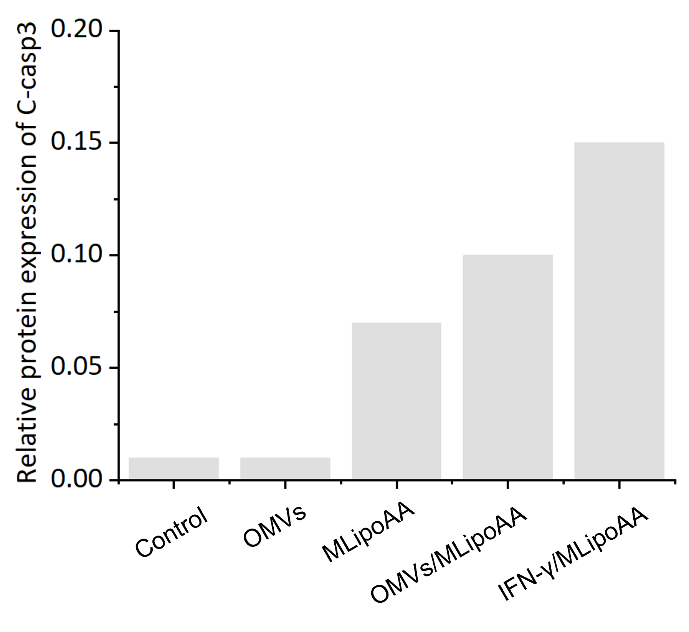


**Figure S25.** Western blot analysis of cleaved caspase-3 proteins in tumor tissues of mice from different groups


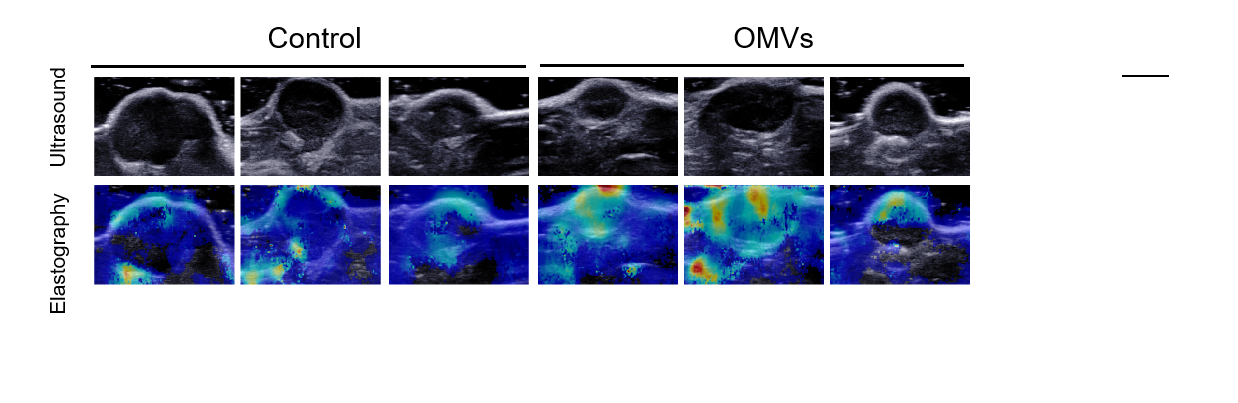


**Figure S26.** In vivo ultrasound images and elastography images of tumor xenografts of mice from control and OMVs treatment groups.


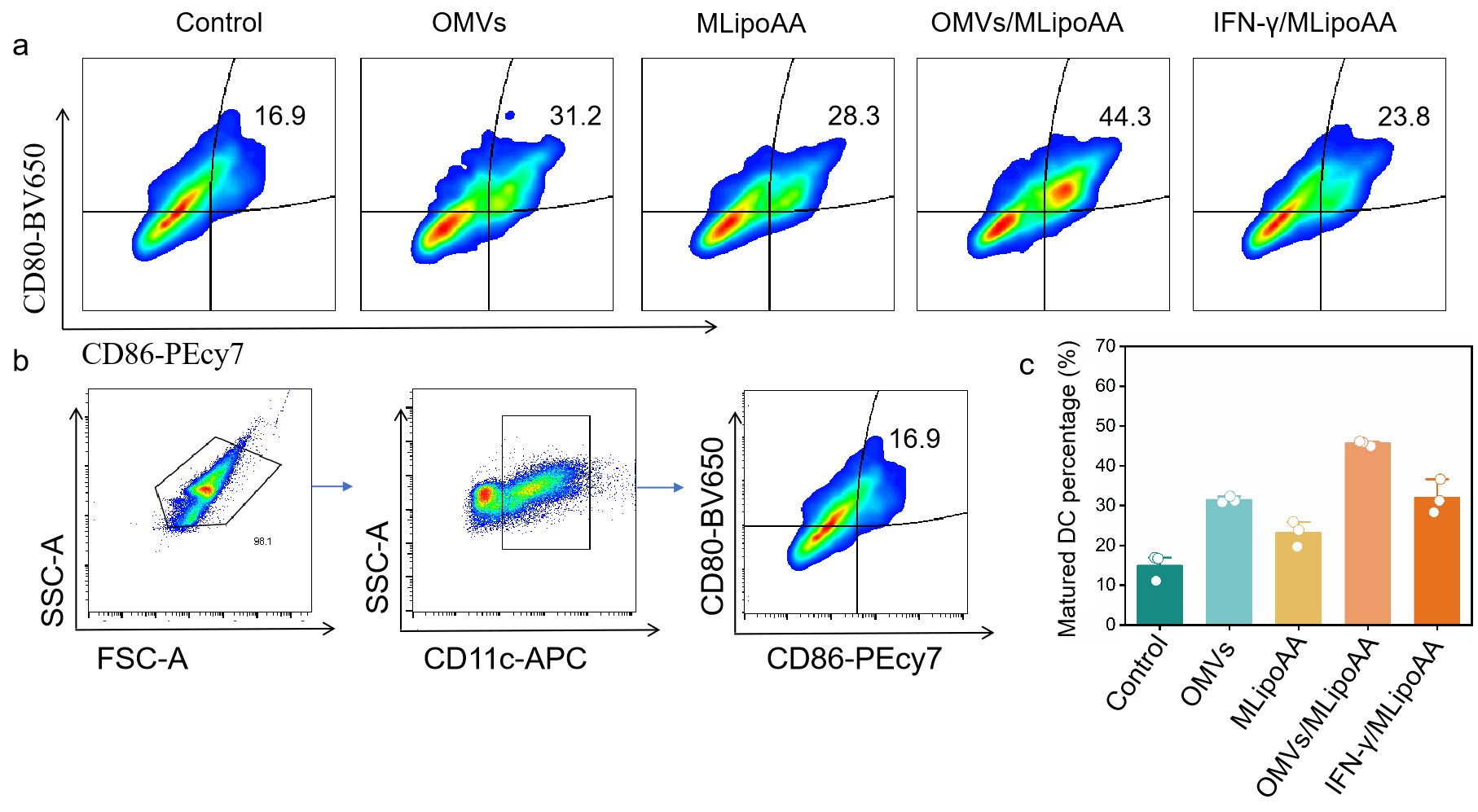


**Figure S27.** (a) Flow cytometry analysis of the percentages of mature DCs (CD80+CD86+ gated on CD11c+) of mice in each group. (b) Gating strategies for the analysis of DCs subsets from lymphocytes. (c) Quantitative analysis of CD80+CD86+ DCs percentage in CD11c+ DCs.


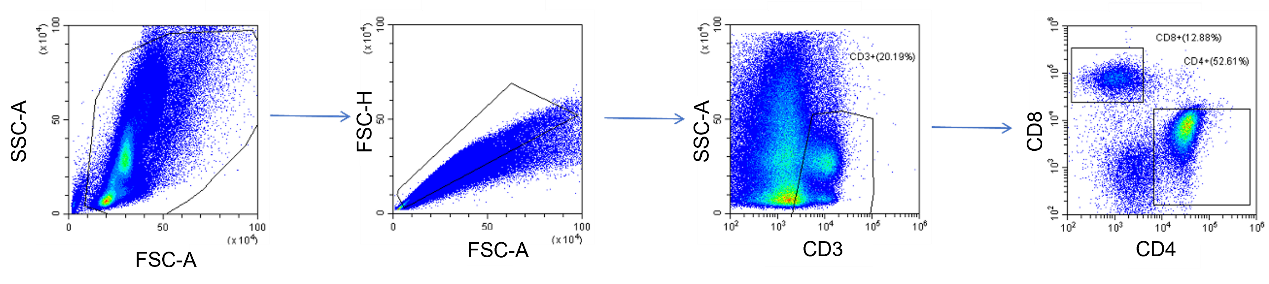


**Figure S28.** Representative gating strategy of spleen lymphocytes for CD8+ T cell assay.


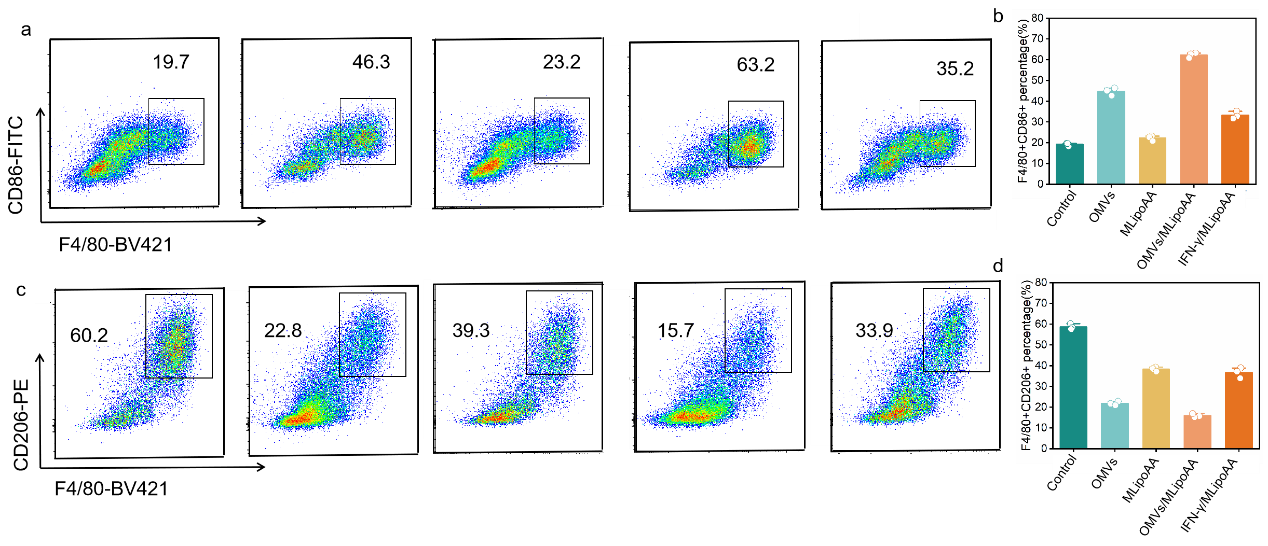


**Figure S29.** Representative flow cytometry analysis of tumor-infiltrating immune cells marked with CD86 and CD206.


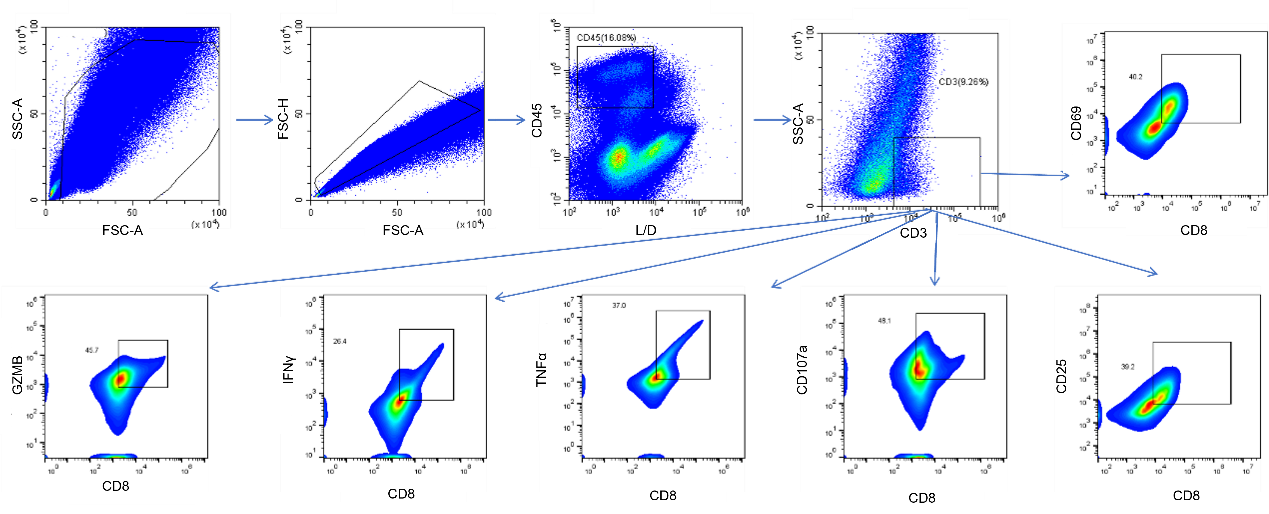


**Figure S30.** Gating strategies for the analysis of the proportions of CD4+ T cells and CD8+T cells with different phenotypic cell states from CT26 tumor tissues.


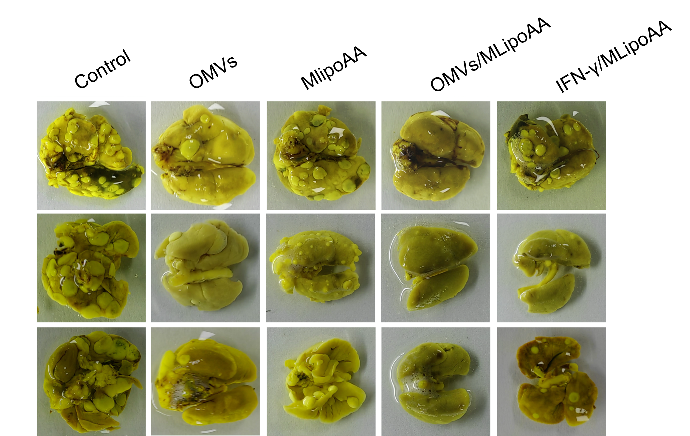

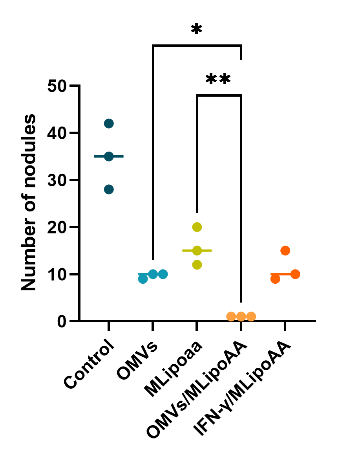


**Figure S31.** Digital photographs and statistical analysis of nodule numbers in lungs after treatments of saline (Control), OMVs, MLipoAA, OMVs/MLipoAA, and IFN-γ/MLipoAA.


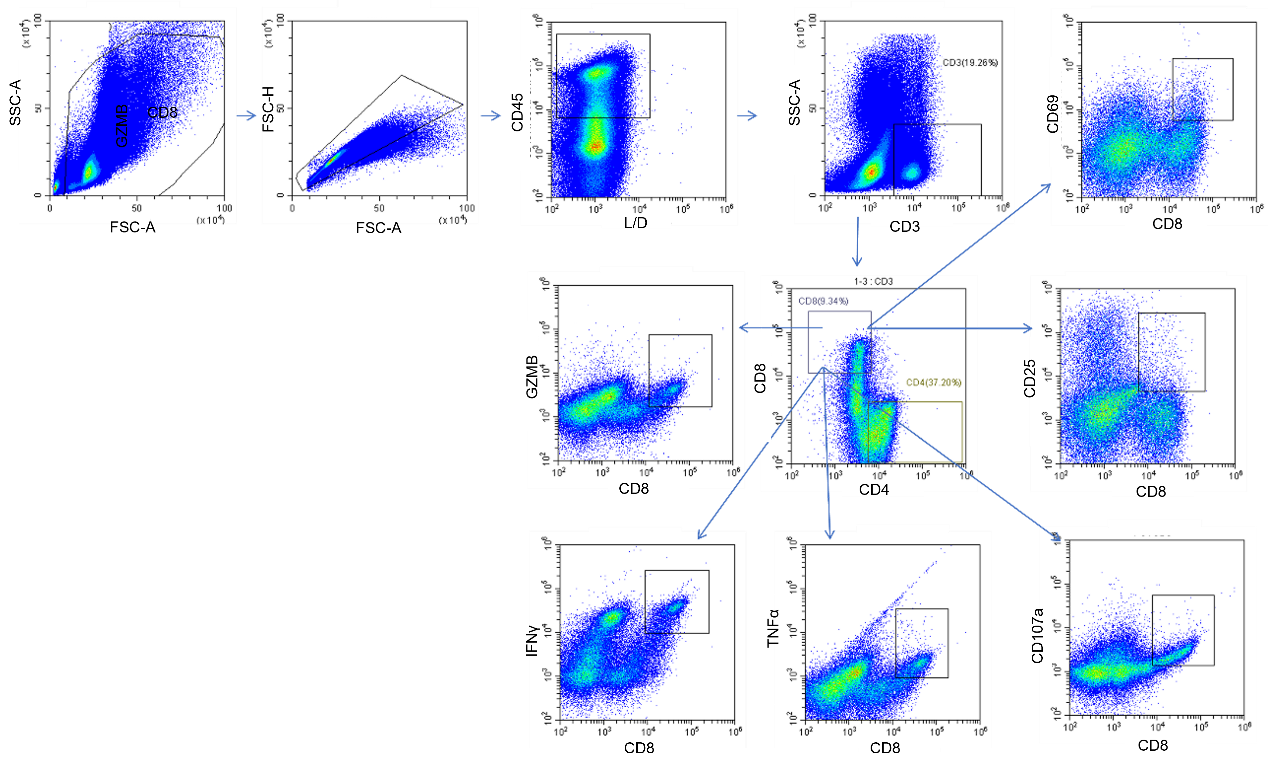


**Figure S32.** Gating strategies for the analysis of the proportions of CD4+ T cells and CD8+T cells with different phenotypic cell states from spleen tissues.


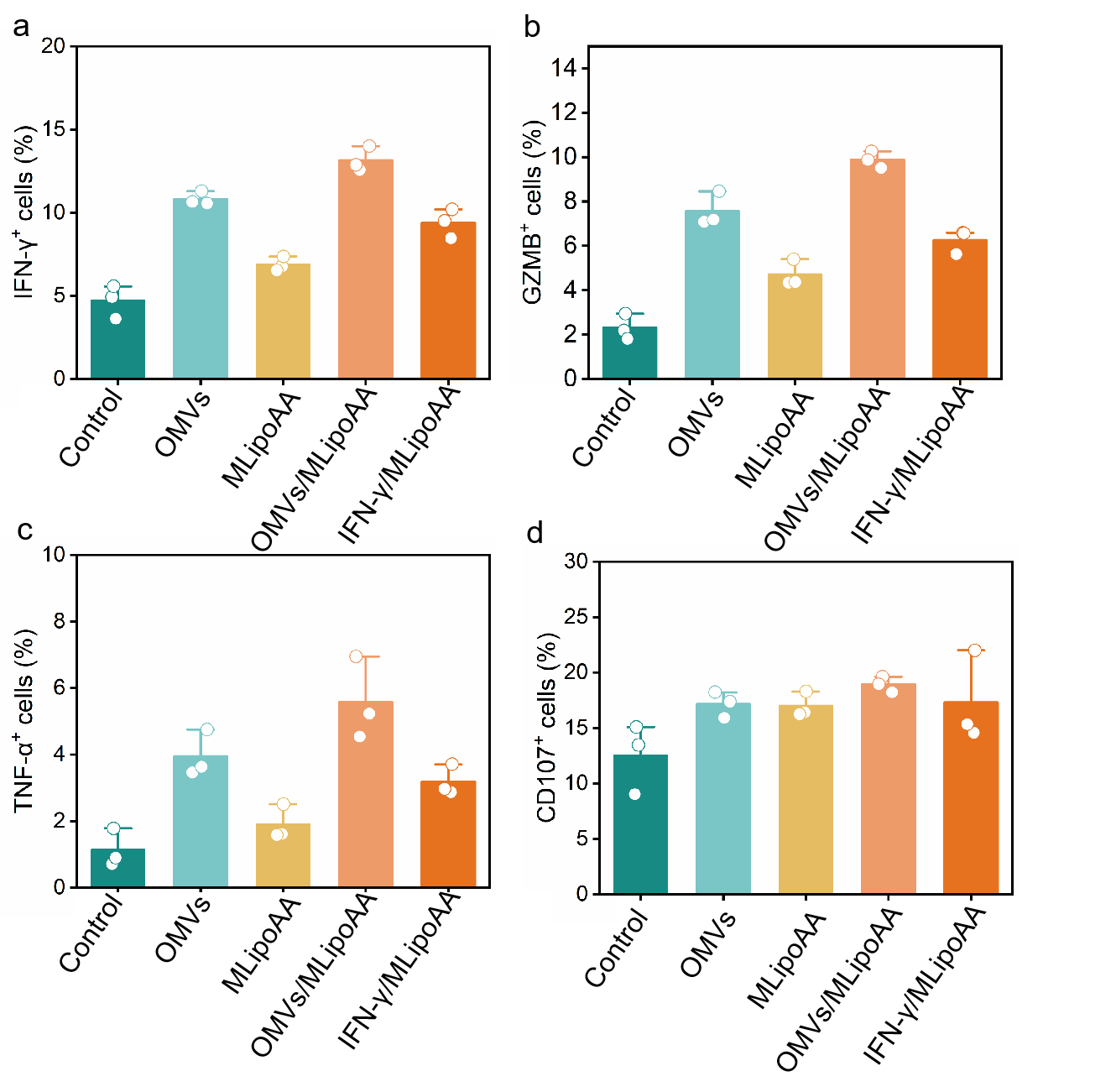


**Figure S33.** Statistical analysis of IFN-γ+CD8+ T cells, GzmB+CD8+ T cells, TNF-α+CD8+ T cells, and CD107+CD8+ T cells of mice from different groups.
